# Effects of dexmedetomidine on early postoperative cognitive function and postoperative inflammatory response: a systematic review and network meta-analysis

**DOI:** 10.3389/fneur.2024.1422049

**Published:** 2024-08-12

**Authors:** Cuifang Huang, Ruimin Yang, Xianlong Xie, Huijun Dai, Linghui Pan

**Affiliations:** ^1^Department of Anesthesiology, Guangxi Medical University Cancer Hospital, Nanning, China; ^2^Guangxi Clinical Research Center for Anesthesiology (GKAD22035214), Nanning, China; ^3^Guangxi Engineering Research Center for Tissue and Organ Injury and Repair Medicine, Nanning, China; ^4^Guangxi Health Commission Key Laboratory of Basic Science and Prevention of Perioperative Organ Dysfunction, Nanning, China; ^5^Department of Anesthesiology, First Affiliated Hospital of Guangxi Medical University, Nanning, China

**Keywords:** dexmedetomidine, postoperative cognitive complications, postoperative cognitive disorder, inflammation, systematic review, network meta-analysis

## Abstract

**Background:**

Dexmedetomidine (DEX) has demonstrated potential as an effective agent for enhancing early postoperative cognitive function. However, there is ongoing debate regarding its optimal dosage and impact on early postoperative inflammatory response. This study aimed to assess and prioritize the effects of varying doses of DEX on early postoperative cognitive function and inflammatory response, in order to identify the most effective intervention dosage.

**Methods:**

Randomised controlled trials (RCTs) and retrospective cohort studies (RCS) from PubMed, Embase, and Cochrane Library up to January 28, 2024, were included. The Mini-Mental State Examination (MMSE) was utilized to assess the impact of varying doses of DEX on cognitive function during the early postoperative period as the primary outcome, peripheral blood levels of IL-6 and TNF-α were considered as secondary outcomes. Meta-analysis and Bayesian Network Meta-Analysis (NMA) were conducted using R. Funnel plots were generated using Stata 15.0.

**Results:**

A total of 29 studies involving 2,807 patients and 25 different doses of DEX were included. DEX was given at a loading dose of 0.3–1.0 μg/kg followed by a maintenance dose of 0.1–0.5 μg/kg/h, or at a uniform intraoperative dose of 0.4–0.7 μg/kg/h. Network meta-analysis revealed most doses of DEX were significantly more effective than normal saline (NS) in improving postoperative MMSE scores (on days 1, 3, and 7) and lowering IL-6 and TNF-α levels. Probability results showed that a 1 μg/kg loading dose followed by a 0.6 μg/kg/h maintenance dose was the best dosing regimen for improving MMSE scores on postoperative days 1 (97.3%), 3 (100%), and 7 (99.9%), as well as for reducing postoperative blood IL-6 levels (1.3%). On the other hand, 0.3 μg/kg followed by 0.2 μg/kg/h was the optimal dosing regimen for reducing postoperative blood TNF-α levels (6.6%).

**Conclusion:**

Compared with NS, intraoperative intravenous DEX improved early postoperative cognitive function and postoperative inflammatory response in patients undergoing elective surgery. In particular, a 1 μg/kg loading dose and a 0.6 μg/kg/h maintenance dose resulted in the best improvement in postoperative MMSE scores and blood IL-6 levels, while a 0.3 μg/kg loading dose followed by a 0.2 μg/kg/h maintenance dose is the optimal regimen for lowering postoperative blood TNF-α levels.

**Systematic review registration**: https://www.crd.york.ac.uk/PROSPERO/display_record.php?RecordID=433932, identifier CRD42023433932.

## 1 Introduction

Abnormalities in postoperative cognitive function can manifest in the anaesthesia recovery room, during hospitalization, or up to 1 year after surgery. These abnormalities may include memory loss, poor concentration, and delayed thinking. Notably, postoperative cognitive abnormalities can result in long-term cognitive impairment, extended hospital stays, increased mortality, and negative socioeconomic consequences. The efficacy and safety of dexmedetomidine (DEX) in treating postoperative cognitive abnormalities and inflammatory response have been supported by numerous randomised, double-blind, placebo-controlled trials (1, 2). DEX has been shown to impact cognitive function and inflammatory outcomes through various mechanisms. Zhao et al. (3) found that DEX inhibited PSD95-NMDA receptor interactions in mice with traumatic brain injury (TBI), leading to improved functional recovery after TBI. Chen et al. (4) reported that DEX enhanced mitochondrial autophagy via PINK1, attenuated hippocampal focal neuroapoptosis, and improved postoperative cognitive dysfunction. Research on epigenetic mechanisms of postoperative cognitive impairment has also indicated that DEX is linked to a lower risk of cognitive decline compared to other substances (5). Kho et al. (6) examined the effects of DEX on autophagic flux, microRNA, and cholinergic anti-inflammatory pathways in LPS-treated rats. The authors revealed that DEX prevented impairment of autophagic flux and reduced apoptosis-associated proteins in the spleen. This suggests that DEX may modulate inflammation through multiple pathways, potentially contributing to its cognitive benefits (6). Furthermore, DEX has been found to play an important role in improving neuroinflammation-induced cognitive dysfunction. Recent studies indicate that DEX ameliorates postoperative cognitive dysfunction (POCD) by activating the c-Jun N-terminal kinase (JNK)/p-38 signaling pathway through CEBPB as a pharmacological target to inhibit M1-mediated inflammation in CNS microglia (7). In a retrospective propensity score study involving patients undergoing lobectomy, DEX prevented cognitive dysfunction and delirium by attenuating neuroinflammation (8). Additionally, a meta-analysis of 17 randomised-controlled trials (RCTs) with 1,619 patients demonstrated that DEX not only ameliorates perioperative immune dysfunction but also reduces POCD and associated neuroinflammation. These findings demonstrate that DEX can enhance postoperative recovery and clinical outcomes in patients undergoing gastrointestinal cancer surgery (9). Clinical guidelines recommend intraoperative DEX infusion for high-risk patients or continuous postoperative infusion for ICU patients to reduce postoperative cognitive abnormalities. However, high doses of DEX as an anaesthetic adjuvant to general intravenous anaesthesia can lower blood pressure and heart rate due to its sympathetic effects. Recovery from these effects may be slow and persistent even after general anaesthesia. Furthermore, there is limited research directly comparing the effects of different DEX doses on postoperative cognitive function and inflammation.

We therefore performed network meta-analyses (NMA) of different doses of DEX. NMA is an extension of traditional meta-analysis, a technique that combines direct and indirect evidence in a network of trials comparing multiple treatments or different doses. In the absence of head-to-head trials, these indirect comparisons provided insights into the optimal dose of DEX to mitigate postoperative cognitive abnormalities and early inflammatory responses.

## 2 Methods

The analyses were conducted in accordance with a pre-specified protocol registered in PROSPERO (CRD42023433932), and the results were reported following the Preferred Reporting Items for Systematic Evaluation and Meta-Analysis (PRISMA) extension statement for NMA (10).

### 2.1 Search strategy and eligibility criteria

Relevant studies were systematically search in Embase, Pubmed, and the Cochrane Library from inception to January 28, 2024 using free-text keywords and MeSH related to DEX and postoperative cognitive disorder without language restrictions (see Appendix A). The retrieved articles were identified and screened by two independent reviewers (RMY and XLX). The relevant procedures for systematic appraisal were in accordance with the PRISMA guidelines. The articles were further selected to assess the expression of postoperative inflammatory markers. The inclusion criteria for this study were: (1) Prospective or retrospective studies of patients undergoing elective surgery with intraoperative intravenous DEX; (2) Patients received general anesthesia, intrathecal anesthesia, nerve block, or other anesthesia modalities; (3) Control group received intraoperative intravenous saline equivalents; (4) Outcomes measured were postoperative MMSE scores and/or postoperative blood levels of IL-6 and/or TNF-α. Studies with the following characteristics were excluded: (1) Lacked a detailed description of the experimental protocol; (2) Abstracts or conference proceedings; (3) Unavailable or untranslated relevant data or unreasonable or seriously flawed study designs.

### 2.2 Data extraction

Data were extracted independently by two authors (CFH, XLX) and entered into a standardized Excel spreadsheet (Microsoft, Redmond, WA, United States). In cases where trials were published in languages other than English, Google Translate was utilized. The extracted trial characteristics included the number of patients in each study group, age, surgical procedure, anesthesia, DEX dose, usage, and duration. The primary endpoints were postoperative MMSE scores on postoperative days 1, 3, and 7, and the secondary endpoints were postoperative blood IL-6 and TNF-α levels. The results were extracted as means and standard deviations. Data presented in graphical form were converted to numerical format using Plot Digitizer (version 2.1, Free Software Foundation, Boston, MA, United States). The median was assumed to be equal to the mean, and the standard deviation was calculated based on the interquartile spacing and extreme deviation (1.35 and 4, respectively) (11). In cases where information was unclear or incomplete, clarification was sought by sending an email to the authors of the included trials.

The quality of all included studies was independently assessed by the investigators (RMY and HJD) according to the Cochrane risk-of-bias tool for randomized trials (RoB2) and Newcastle-Ottawa Scale (NOS). The quality of randomized-controlled trials (RCTs) was assessed using the RoB2 tool in five domains, namely randomization process, deviation from intended intervention, missing outcome data, measurement of outcomes, and selection of reported outcomes. Each domain was scored low risk, some level of concern, or high risk. Any disagreements were resolved through discussion. The NOS scale was used to rate the quality of retrospective cohort studies (RCS), with scores ranging from 1 to 3 indicating low quality, 4 to 6 indicating moderate quality, and 7 to 9 indicating high quality.

### 2.3 Statistical analysis

Meta-analysis and Bayesian NMA were performed using the ‘gemtc’ and ‘rjags’ packages of R v4.3.1 (12). Network graphs were generated for each outcome displaying different DEX doses as nodes and direct comparisons as connecting lines. Indirect comparisons were derived from direct comparison estimates of a common comparison dose, and the results of both direct and indirect comparisons were summarized in a network leaderboard. MMSE scores (postoperative days 1, 3, 7) and postoperative blood IL-6 and TNF-α levels are expressed as ratios (OR) and 95% confidence intervals (CI) (13). The advantages and disadvantages of different DEX doses were ranked according to the surface under the cumulative ranking curve (SUCRA). The intervention dose is absolutely effective if SUCRA is ≥0 and ≤ 1, and absolutely ineffective if SUCRA is 0. For IL-6 and TNF-α, a larger area under the curve (AUC) indicates a more detrimental effect of postoperative inflammatory markers on recovery. Heterogeneity among the results of the included studies was determined by the χ^2^ test (significance level *α* = 0.1) and quantitated by *I^2^*. The fixed effects model was used if there was no heterogeneity between studies (*I^2^* ≤ 50%); otherwise, a random effects model was used (*I^2^* > 50%). Significant clinical heterogeneity was addressed through methods such as subgroup analysis, sensitivity analysis, or descriptive analysis only.

The robustness of results can be influenced by the size of the study and the likelihood of publishing negative results. In addition, we conducted one-way sensitivity analyses to evaluate the influence of the included studies on the combined results, considering the significantly heterogeneous outcomes. Funnel plots were created using Stata version 15.0 (Stata Corp, College Station, TX, United Sates), and Egger’s test were conducted to visually assess publication bias for outcomes that included 10 or more studies.

## 3 Results

### 3.1 Characteristics of included studies

**Figure 1:**
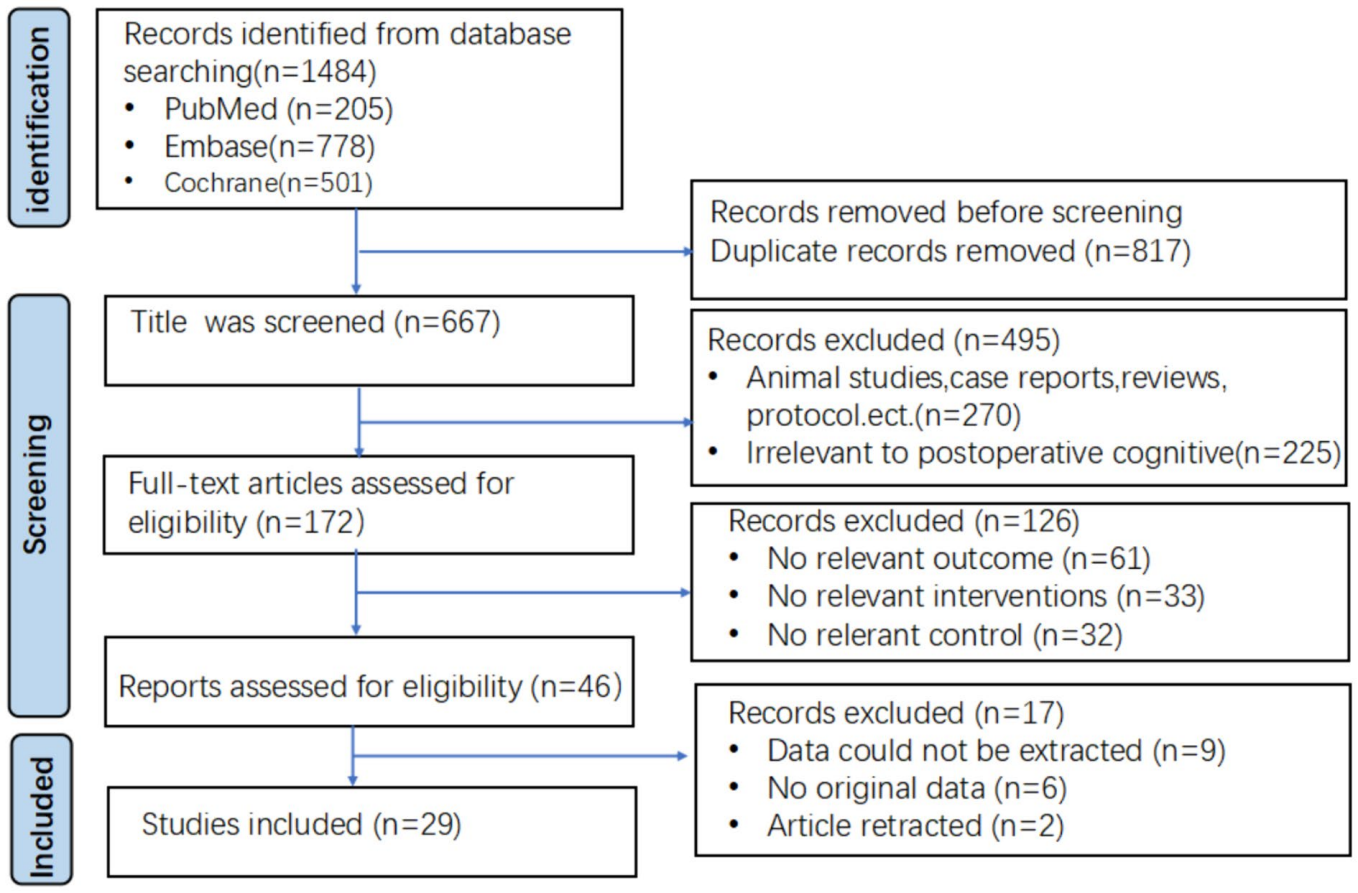
Flow chart of the study selection procedure.

A total of 1,484 relevant articles were collected from the three databases. After removing duplications, the title of the remaining 667 articles was screened by two independent reviewers and 495 studies were excluded due to inappropriate study type, including animal studies, case reports, reviews, protocols, and studies unrelated to postoperative cognition. Upon further examination of the abstracts, 126 studies were excluded due to failure to meet the inclusion criteria, including 61 with unrelated study outcomes, 33 with unrelated study interventions, and 32 with unrelated study controls. Following a thorough review of the full text, 17 studies were eliminated, comprising 9 articles with unextractable data, 6 articles lacking raw data, and 2 retracted articles. Ultimately, 29 studies involving 2,807 patients were included. The included studies had comparable baseline characteristics, such as sex, age, and sample size. The screening process is depicted in Figure 1.

Of these 29 studies, 23 RCTs and 3 RCSs reported postoperative cognitive function outcomes (MMSE), 10 RCTs and 1 RCS reported postoperative blood IL-6 expression, and 8 RCTs and 1 RCS reported postoperative blood TNF-α level. The mean age of the participants ranged from 40 to 75 years (Table 1). Most trials were placebo-controlled (89%) and had two treatment groups (72.4%). The endpoints assessed were postoperative cognitive function (assessed by MMSE) and postoperative inflammatory markers (IL-6 and TNF-α). General anesthesia was used in 25 studies, nerve block in 1 study, epidural block in 2 studies, and lumbar nerve block in 1 study. MMSE was assessed at 3 time points, namely postoperative days 1 (*n* = 20), 3 (*n* = 13), and 7 (*n* = 10), with postoperative day 1 being the predominant time point. Inflammatory markers (IL-6 and TNF-α) were assessed at 1 day or 1 h post-surgery, most commonly on postoperative day 1. A total of 24 DEX doses were identified. Four studies (16, 17, 23, 34) used a 1 μg/kg loading dose (LD) followed by a 0.4 μg/kg/h maintenance dose (MD); two studies (15, 42) used 1 μg/kg LD then 0.3 μg/kg/h MD; two studies (18, 29) used 0.5 μg/kg LD then 0.2 μg/kg/h MD; two studies (25, 26) used 0.3 μg/kg LD then 0.2 μg/kg/h MD; two studies (25, 26) used 0.3 μg/kg LD then 0.5 μg/kg/h MD; two studies (25, 26) used 0.3 μg/kg LD then 0.8 μg/kg/h MD; two studies (28, 31) used an intraoperative dose (IOD) of 0.5 μg/kg/h; two studies (30, 33) used 0.4 μg/kg/h IOD; two studies (36, 40) used 0.6 μg/kg/h IOD; one study (14) used 0.7 μg/mL IOD; one study (19) used 0.6 μg/kg LD then 0.2 μg/kg/h MD; one study (20) used 1 μg/kg LD then 0.3–0.5 μg/kg/h MD; one study (21) used 0.3 μg/kg LD then 0.3 μg/kg/h MD; one study (22) used 1 μg/kg LD then 0.2 μg/kg/h MD; one study (24) used 0.5 μg/kg LD then 0.1 μg/kg/h MD; one study (27) used 0.5 μg/kg LD then 0.6 μg/kg/h MD; one study (41) used 0.8 μg/kg LD then 0.5 μg/kg/h MD; one study (32) used 1 μg/kg LD then 0.5 μg/kg/h MD; one study (35) used 0.6 μg/kg LD only; one study (37) used 0.5 μg/kg for 30 min before the end of surgery; one study (38) used 0.5 μg/kg LD then 0.4 μg/kg/h MD; one study (39) used 0.8 μg/kg LD then 0.2 μg/kg/h MD; one study (40) used 0.3 μg/kg/h IOD; and one study (42) used 1 μg/kg LD then 0.6 μg/kg/h MD.

**Table 1:** Characteristics of the included trials.

**Study (year)**	**Country**	**Sample size**	**Comparison**	**Age, y**	**Type of surgery**	**Anaesthetic method**	**Dosage**	**Assessment methods**	**Secondary outcomes**
Ao et al. (2022) (14)	Germany	60/60	DEX vs NS	57.64 ± 11.35/58.80 ± 12.20	Lower extremity fractures	Nerve block	0.7 μg/mL IOD	MMSE	/
Chen et al (2020) (15)	China	43/45	DEX vs NS	64.9 ± 11.4/65.4 ± 11.7	Colorectal cancer	GA	1 μg/kg LD then 0.3 μg/kg/h MD	MMSE	/
Chen et al (2013) (16)	China	63/63	DEX vs NS	66.2 ± 7.5/67.9 ± 6.6	Laparoscopic cholecystectomy	GA	1 μg/kg LD then 0.4 μg/kg/h MD	MMSE	/
Chen et al. (2021) (17)	China	40/40	DEX vs NS	50.76 ± 8.32 /51.07 ± 9.43	Intestinal surgery	GA	1 μg/kg LD then 0.4 μg/kg/h MD	MMSE	IL-6, TNF-α
Chen et al (2015) (18)	China	87/61	DEX vs NS	/	Mixed	GA	0.5 μg/kg LD then 0.2 μg/kg/h MD	MMSE	IL-6, TNF-α
Gao et al. (2021) (19)	China	20/20	DEX vs NS	70.5 ± 4.1 / 71.4 ± 4.5	Elective minimally invasive off-pump coronary artery bypass grafting	GA	0.6 μg/kg LD then 0.2 μg/kg/h MD	MMSE	/
Gao et al. (2020) (20)	China	30/30	DEX vs NS	69.5 ± 5.1/70.4 ± 4.2	Elective minimally invasive off-pump coronary artery bypass grafting	GA	1 μg/kg LD then 0.3-0.5 μg/kg/h MD	MMSE	/
Ge et al (2019) (21)	China	25/24	DEX vs NS	70 ± 3/72 ± 5	Carotid endarterectomy	GA	0.3 μg/kg LD then 0.3 μg/kg/h MD	MMSE	IL-6, TNF-α
Gong et al. (2018) (22)	China	40/40	DEX vs NS	42.3 ± 1.6 /42.4 ± 1.5	Extracorporeal coronary artery bypass grafting	GA	1μg/kg LD then 0.2 μg/kg/h	MMSE	/
Li et al (2015) (23)	China	60/60	DEX vs NS	69 ± 5 /70 ± 6	Laparoscopic cholecystectomy	GA	1 μg/kg LD then 0.4 μg/kg/h MD	MMSE	IL-6
Li et al. (2020) (24)	China	41/46	DEX vs NS	67.37 ± 3.27 /67.26 ± 2.07	Lung cancer resection	GA	0.5 μg/kg LD then 0.1 μg/kg/h MD	MMSE	/
Li et al. (2021) (25)	China	30/30/30/30	DEX1 vs DEX2 vs DEX3 vs NS	74.7 ± 2.6 /71.2 ± 3.5 /69.8 ± 4.3 /73.4 ± 5.1	Spinal surgery	GA	0.3 μg/kg LD then 0.2, 0.5, and 0.8 μg·kg-1/h MD	MMSE	IL-6, TNF-α
Li et al. (2021) (26)	China	20/20/20/20	DEX1 vs DEX2 vs DEX3 vs NS	74.7 ± 2.6 /71.2 ± 3.5 /69.8 ± 4.3 /73.4 ± 5.1	/	GA	0.3 μg/kg LD then 0.2, 0.5, and 0.8 μg/kg/h MD	MMSE	IL-6, TNF-α
Liu et al. (2020) (27)	China	24/24/24/24	DEX vs DEX+epidural blockade) vs NS	69.6 ± 4.4/69.3 ± 4.4 /68.5 ± 4.2/ 68.6 ± 3.9	Radical resection for colorectal cancer	epidural blockade	0.5 μg/kg LD then 0.6 μg/kg/h MD	MMSE	/
Shi et al. (2020) (28)	China	53/53	DEX vs NS	68.71 ± 4.63 /68.7 ± 3.40	Thoracoscopic lobectomy	GA	0.5 μg/kg/h IOD	MMSE	/
Shi et al. (2020) (29)	China	40/40	DEX vs NS	66.4 ± 5.2/67.6 ± 5.5	Lung cancer	GA	0.5 μg/kg LD then 0.2 μg/kg/h MD	MMSE	/
Wang et al (2022) (30)	China	60/60	DEX vs NS	65.6 ± 3.4 /65.6 ± 3.4	Ureteroscopic holmium laser lithotripsy	GA	0.4 μg/kg/h IOD	MMSE	/
Wang et al (2017) (31)	China	48/48	DEX vs NS	56.43 ± 6.57/56.38 ± 6.47	Heart valve replacement surgery	GA	0.5 μg/kg/h IOD	MMSE	/
Zhu et al (2021) (32)	China	95/92	DEX vs NS	74.1 ± 4.36 /75.25 ± 6.10	Orthopedic surgery	epidural blockade	1 μg/kg LD then 0.5 μg/kg/h MD	MMSE	/
Zhou et al. (2019) (33)	China	39/38/39/38	Ulinastatin+ DEX vs ulinastatin vs DEX vs saline	70.6 ± 4.4 /69.8 ± 5.1 /69.8 ± 5.1 /70.0 ± 4.9	Selective laparoscopic sleeve gastrectomy (LSG)	GA	0.4 μg/kg/h IOD	/	IL-6, TNF-α
Zhang et al (2021) (34)	China	87/87	DEX vs NS	70.6 ± 4.2/71.4 ± 4.9	Mixed	GA	1 μg/kg LD then 0.4 μg/kg/h MD	MMSE	IL-6, TNF-α
Xu et al (2020) (35)	China	100/86	DEX vs NS	62.9 ± 7.8 /61.4 ± 7.3	Hip replacement	Lumbar nerve block	0.6 μg/kg for 15 min before anesthesia induction	MMSE	IL-6, TNF-α
Du et al (2019) (36)	China	20/20/20/20	Parecoxib vs Dexmedetomidine vs Combined vs NS	67.2 ± 11.4/ 69.3 ± 12.5 /70.4 ± 14.7 / 68.7 ± 13.5	Laparoscopic cholecystectomy surgery	GA	0.6 μg/kg/h IOD	MMSE	/
Ma et al (2017) (37)	China	25/25/25/25	FA vs Dex vs Dex +FA vs NS	47.13 ± 7.25 /49.28 ± 9.33 /47.95 ± 8.36 /48.07 ± 9.19	Thyroid surgery	GA	0.5 μg/kg LD	MMSE	/
Wang et al (2020) (38)	China	60/50	DEX vs NS	68.37 ± 3.27 /68.26 ± 2.07	Radical gastrectomy	GA	0.5 μg/kg LD then 0.4 μg/kg/h MD	MMSE	/
Liu et al (2020) (39)	China	30/30	DEX vs NS	43.4 ± 8.9 / 40.6 ± 9.4	Sleep apnea syndrome	GA	0.8 μg/kg LD then 0.2 μg/kg/h MD	MMSE	/
Tang et al (2022) (40)	China	40/40/40	DEX1 vs DEX2 vs NS	68.71 ± 6.64 /66.27 ± 7.16 /69.67 ± 6.87	Hepaticlobectomy	GA	0.3 μg/kg/h or 0.6 μg/kg/h after anesthesia induction	/	TNF-α
Xu et al. (2017) (41)	China	48/48	DEX vs NS	71.89 ± 31.36/72.06 ± 32.17	Laparoscopic ovarian cystectomy	GA	0.8 μg/kg LD then 0.5 μg/kg/h MD	/	IL-6
Li et al. (2018) (42)	China	20/20/20	DEX1 vs DEX2 vs NS	65.8 ± 4.28/66.3 ± 3.54/66.1 ± 3.96	Femaral head replacement	GA	1 μg/kg LD then 0.3 μg/kg/h or 0.6μg/kg/h MD	MMSE	IL-6

DEX, Dexmedetomidine; NS, Normal saline; GA, General anesthesia; MMSE, MiniiMental State Examination; LD, Loading dose; MD, Maintenance dose; IOD, Intraoperative uniform dose.

### 3.2 Results of risk of bias, heterogeneity and sensitivity analysis

**Figure 2:**
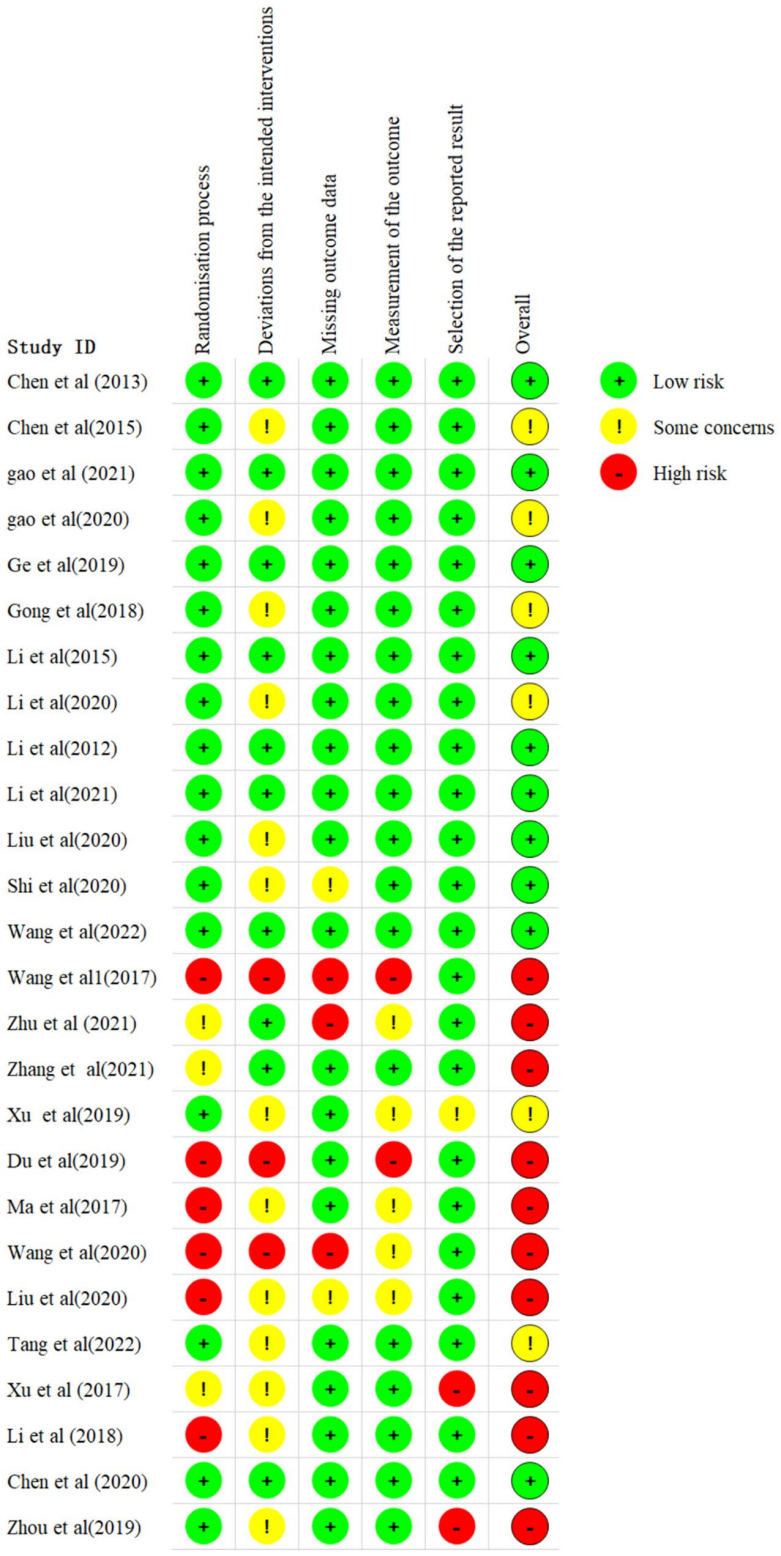
Assessments of every risk of bias item for eligible studies. The green, yellow, and red circle indicate “low, ““unclear,” and “high” risk of bias, respectively.

Of the 26 RCTs, 17 (65.3%) utilized a method to generate a randomized sequence of appropriate methods, 3 (11.5%) deviated from the intervention, and 3 (11.5%) experienced loss of data. Additionally, 19 (26.9%) RCTs had outcome measurement bias and 2 (7.6%) reported selection bias in the outcome. Quality assessment of the included studies revealed that 38.5% were of low risk, 23.1% were of unknown risk of bias, and 38.5% were of high risk (Figure 2). The overall quality of the included studies ranged from low to moderate. In addition, quality assessment of the 3 RCSs using the NOS scale showed that they were all high-quality (Table 2).

**Table 2:** Newcastle-Ottawa Scale.

	Selection of cohorts					Outcome			
Selection of cohorts	Representativeness of the exposed cohort	Selection of the non-Exposed cohort	Ascertainment of exposure	Demonstration that outcome of interest was not present at start of study	Comparability of cohorts	Assessment of outcome	Was follow-up long enough for outcomes to occur	Adequacy of follow-up of cohorts	Overall
Ao et al. (14)	★	★	★	★	★	★		★	7
Chen et al. (18)	★	★	★	★	★	★		★	7
Shi et al. (29)	★	★	★	★	★★	★	★	★	9

★: appropriate study design; ★★: one for the most important factor; the other for another factor.

The included studies were heterogeneous (*I^2^*>50%), except for the MMSE scores on postoperative days 3 and 7 (Supplementary Figures 1a–e). The heterogeneity test indicated an *I^2^* value greater than 50%, with no significant variations observed in study design, region, participant characteristics, interventions, or methodology. Subsequent sensitivity analyses identified the Li et al. (25) and Li et al. (26) studies as the primary sources of heterogeneity (Supplementary Figure 1). Upon re-evaluation, it was discerned that both studies exhibited bias due to small sample sizes in each group. After excluding these studies, the *I^2^* value dropped below 50%, leading to enhanced consistency and stability in the results of the net meta-analysis.

The pooled results of our network meta-analyses may have been influenced by several confounding factors, including the type of surgery, age, and method of assessment. To ensure the reliability of our findings, we performed a series of sensitivity analyses. The results of these analyses, presented in Supplementary Figures 2a–e, did not reveal any significant changes in the pooled results for all comparisons.

### 3.3 Primary outcome: MMSE scores on postoperative days 1, 3 and 7

**Figure 3:**
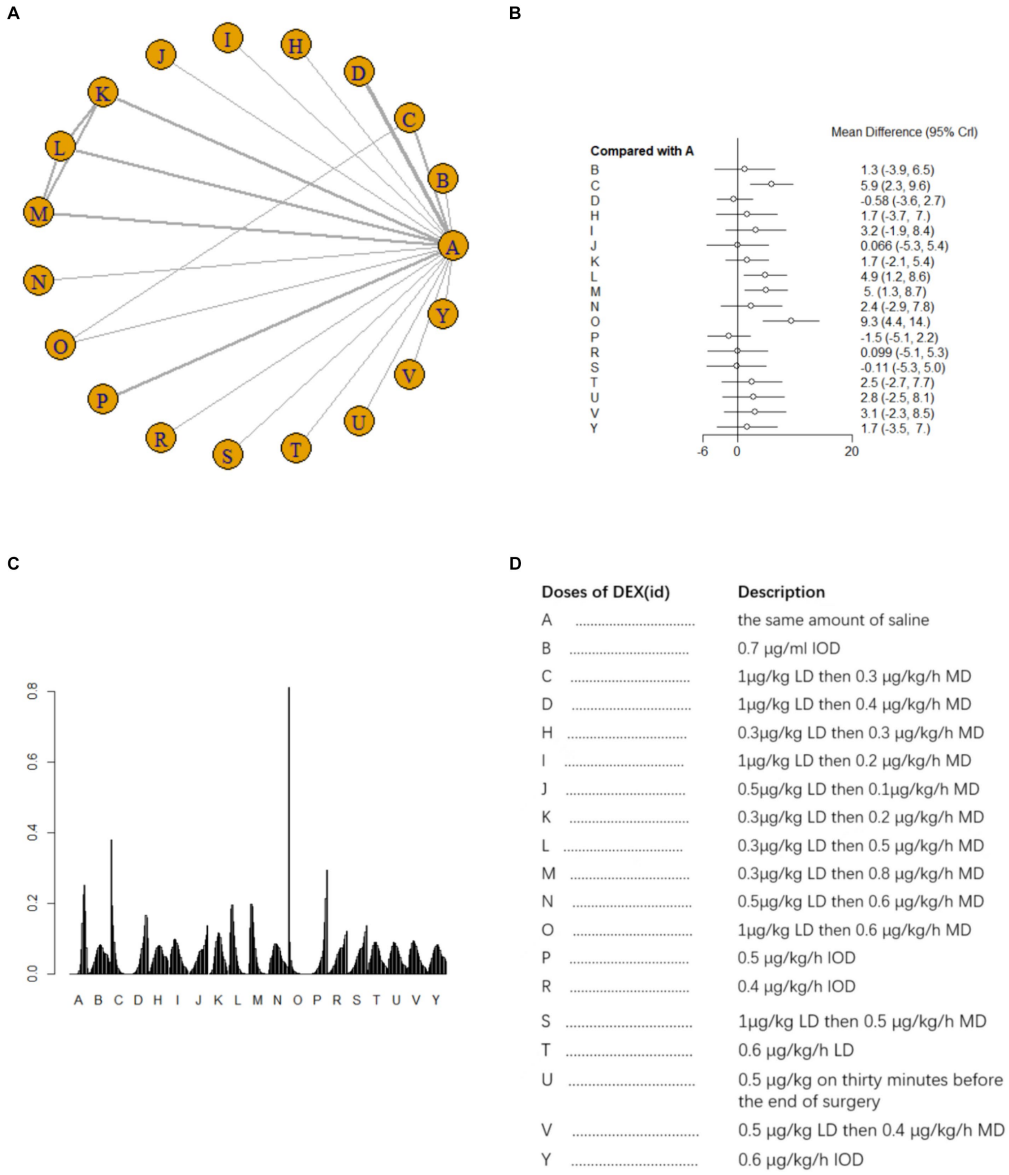
Network estimates of total MMSE score on day 1 after surgery. **(A)** Network plot showing direct comparisons between nodes. **(B)** Forest plot of different DEX doses compared with NS in NMA. **(C)** Estimated probability (%) of each dose level. **(D)** Description of individual DEX doses in this analysis. LD, Load dose; MD, Maintenance dose; IOD, Intraoperative dose.

A total of 20 studies (14, 15, 17, 21–28, 30–32, 34–38, 42) involving 2041 patients assessed MMSE scores on postoperative day 1. There was heterogeneity among the studies (*I^2^* = 84%) and a random effects model was used for meta-analysis. A total of 18 intervention doses were involved in the NMA, and the evidence relationships are shown in Figure 3A. Compared with the placebo, four DEX dosing regimens (1 μg/kg LD followed by 0.3 μg/kg/h MD, 0.3 μg/kg LD followed by 0.5 μg/kg/h MD, 0.3 μg/kg LD followed by 0.8 μg/kg/h MD, and 1 μg/kg LD followed by 0.6 μg/kg/h MD) significantly improved postoperative MMSE scores (SMD 5.9, 95%CI 2.3 to 9.6; SMD 4.9, 95%CI 1.2 to 8.6; SMD 5, 95%CI 1.3 to 8.7; SMD 9.3, 95%CI 4.4 to 14, respectively) (Figure 3B). When comparing the effects of different DEX doses on improving MMSE scores, 1 μg/kg LD then 0.6 μg/kg/h MD was significantly better than 0.7 μg/mL IOD, 1 μg/kg LD then 0.4 μg/kg/h MD, 0.5 μg/kg LD then 0.1 μg/kg/h MD, 0.5 μg/kg/h IOD, 0.4 μg/kg/h IOD, 1 μg/kg LD then 0.5 μg/kg/h MD and 0.6 μg/kg/h IOD; 1 μg/kg LD then 0.3 μg/kg/h MD was significantly better than 1 μg/kg LD then 0.4 μg/kg/h MD and 0.5 μg/kg/h IOD; 1 μg/kg LD then 0.4 μg/kg/h MD was significantly worse than 0.3 μg/kg LD then 0.5 μg/kg/h MD and 0.3 μg/kg LD then 0.8 μg/kg/h MD (Supplementary Table 1a). The probability of improvement in MMSE scores on postoperative day 1 by different DEX doses versus NS was analyzed by determining the SUCRA (Figure 3C). Refer to Figure 3D for abbreviations and descriptions of the 18 DEX intervention doses and the control group. Our data showed that 1 μg/kg LD then 0.6 μg/kg/h MD resulted in the best MMSE score improvement (97.3%), followed by 1 μg/kg LD then 0.6 μg/kg/h MD (85.1%). An IOD of 0.5 μg/kg/h DEX (13.1%) resulted in the worst MMSE score improvement.

**Figure 4:**
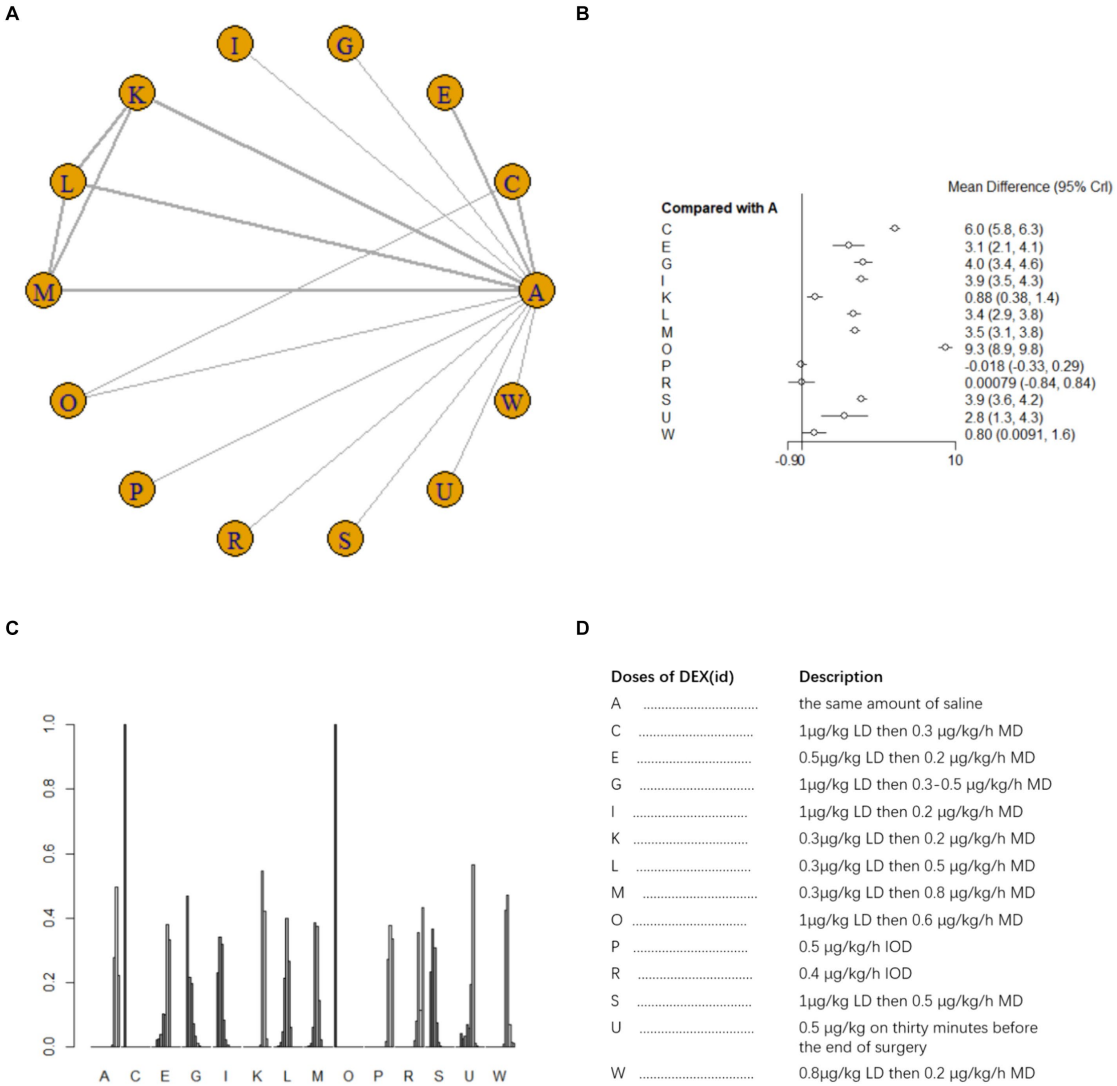
Network estimates of total MMSE score on day 3 after surgery. **(A)** Network plot showing direct comparisons between nodes. **(B)** Forest plot of different DEX doses compared with NS in NMA. **(C)** Estimated probability (%) of each dose level. **(D)** Description of individual DEX doses in this analysis. LD, Load dose; MD, Maintenance dose; IOD, Intraoperative dose.

Thirteen studies (15, 18, 20, 22, 25, 26, 28–30, 32, 37, 39, 42) involving 1,279 patients assessed MMSE scores on postoperative day 3. A fixed effects model was utilized for meta-analysis due to the presence of low heterogeneity (*I^2^* = 23%). A total of 13 intervention doses were involved in the NMA, and the evidence relationships are shown in Figure 4A. All 11 doses of dexmedetomidine significantly improved postoperative MMSE scores compared with placebo, except for two doses (0.4μg/kg/h IOD and 0.5 μg/kg/h IOD) (Figure 4B). In the NMA, there were significant differences among different doses of DEX (Supplementary Table 1b). The cumulative ranking of different DEX doses versus NS for improvement in MMSE score on postoperative day 3 is shown in Figure 4C. Figure 4D for abbreviations and descriptions of the 13 DEX intervention doses and the control group. SUCRA analysis showed that the improvement in MMSE score was the greatest with 1 μg/kg LD then 0.4 μg/kg/h MD (100%), followed by 1 μg/kg LD then 0.3 μg/kg/h, and the least with 0.5 μg/kg/h IOD (7.4%) (Figure 4C).

**Figure 5:**
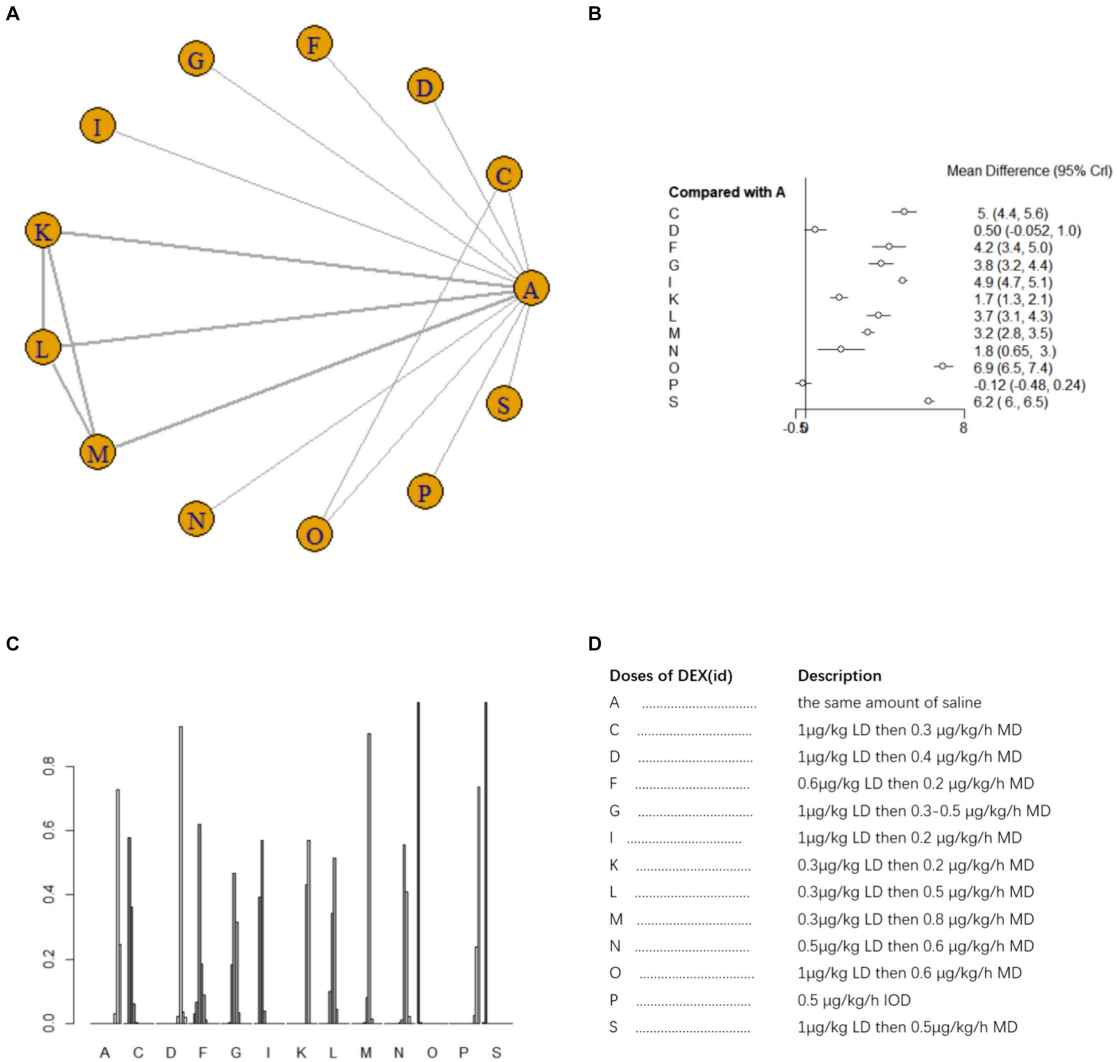
Network estimates of total MMSE score on day 7 after surgery. **(A)** Network plot showing direct comparisons between nodes. **(B)** Forest plot of different DEX doses compared with NS in NMA. **(C)** Estimated probability (%) of each dose level. **(D)** Description of individual DEX doses in this analysis. LD, Load dose; MD, Maintenance dose; IOD, Intraoperative dose.

Ten studies (16, 19, 20, 22, 25–28, 32, 42) involving 950 patients evaluated MMSE scores on postoperative day 7. A fixed effects model was adopted for meta-analysis owing to the small heterogeneity among studies (*I^2^* = 23%). A total of 12 intervention doses were involved in the NMA, and the evidence relationships are shown in Figure 5A. Compared with the placebo, ten DEX dosing regimens (1 μg/kg LD then 0.3 μg/h MD, 0.6 μg/kg LD then 0.2 μg/kg/h MD, 1 μg/kg LD then 0.3–0.5 μg/kg/h MD, 1 μg/kg LD then 0.2 μg/kg/h MD, 0.3 μg/kg LD then 0.2 μg/kg/h MD, 0.3 μg/kg LD then 0.5 μg/kg/h MD, 0.3 μg/kg LD then 0.8 μg/kg/h MD, 0.5 μg/kg LD then 0.6 μg/kg/h MD, 1 μg/kg then 0.5 μg/kg/h MD, 1 μg/kg LD then 0.6 μg/kg/h MD) significantly improved MMSE scores on postoperative day 7 (SMD 5, 95%CI 4.4 to 5.6; SMD 4.2, 95%CI 3.4 to 5.0; SMD 3.8, 95%CI 3.2 to 4.4; SMD 4.9, 95%CI 4.7 to 5.1; SMD 1.7, 95%CI 1.3 to 2.1; SMD 3.7, 95%CI 3.1 to 4.3; SMD 3.2, 95%CI 2.8 to 3.5; SMD 1.8, 95%CI 0.65 to 3; SMD 6.2, 95%CI 6 to 6.5; SMD 6.9, 95%CI 6.5 to 7.4, respectively) (Figure 5B). In the NMA, there were significant differences among different doses of DEX (Supplementary Table 1c). The cumulative ranking of different DEX doses versus NS on the improvement of MMSE on postoperative day 7 is shown in Figure 5C. Figure 5D for abbreviations and descriptions of the 12 DEX intervention doses and the control group. SUCRA analysis revealed that improvement in MMSE scores was the greatest with 1 μg/kg LD then 0.6 μg/kg/h MD (99.9%), followed by 1 μg/kg LD then 0.5 μg/kg/h MD (91.6%) and 1 μg/kg LD then 0.2 μg/kg/h MD (77.9%), and the least with NS (6.4%) and 0.5 μg/kg/h IOD (2.4%) (Figure 5C).

### 3.4 Secondary outcomes: postoperative blood IL-6 and TNF-α levels

**Figure 6:**
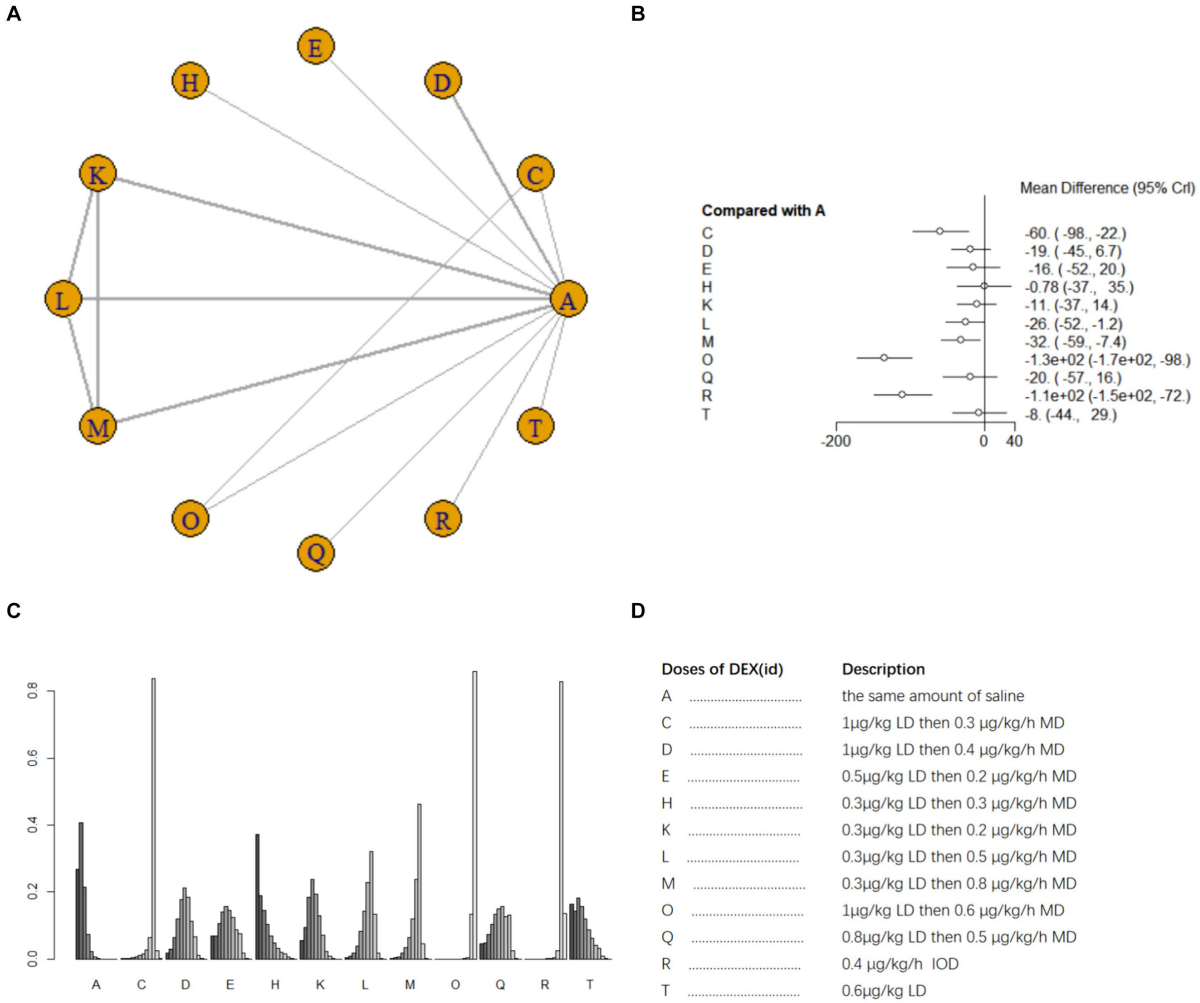
Network estimates of blood IL-6 level after surgery. **(A)** Network plot showing direct comparisons between nodes. **(B)** Forest plot of different DEX doses compared with NS in NMA. **(C)** Estimated probability (%) of each dose level. **(D)** Description of individual DEX doses in this analysis. LD, Load dose; MD, Maintenance dose; IOD, Intraoperative dose.

Ten studies (17, 18, 21, 25, 26, 33–35, 41, 42) involving 1,110 patients examined postoperative IL-6 level. There was high heterogeneity among the studies (*I^2^* = 88%) and a random effects model was utilized. A total of 11 intervention doses were involved in the NMA, and the evidence relationships are shown in Figure 6A. Compared with the placebo, five DEX dosing regimens (1 μg/kg LD then 0.3 μg/kg/h MD, 0.3 μg/kg LD then 0.5 μg/kg/h MD;0.3 μg/kg LD then 0.8 μg/kg/h MD;1 μg/kg LD then 0.6ug/kg/h MD; 0.4 μg/kg/h IOD) significantly decreased postoperative IL-6 level (SMD-60, 95%CI -98 to −22; SMD -26 95%CI -52 to −1.2; SMD -32 95%CI -59 to −7.4; SMD -1.3e+0.2, 95%CI -1.7e to −98; SMD -1.1e+0.2, 95%CI -1.5e+0.2 to −72, respectively) (Figure 6B). Among the different doses, 1 μg/kg LD then 0.6 μg/kg/h led to significantly greater reduction in postoperative blood IL-6 level than most other dosing regimens; 1 μg/kg LD then 0.3 μg/h MD was significantly better than 0.3 μg/kg LD then 0.3 μg/kg/h MD and 0.3 μg/kg LD then 0.2 μg/kg/h MD; 0.4 μg/kg/h IOD was significantly worse than most other dosing regimens (Supplementary Table 1d). The cumulative ranking of different DEX doses versus NS in reducing postoperative blood IL-6 level is shown in Figure 6C, refer to Figure 6D for abbreviations and descriptions of the 11 DEX intervention doses and the control group, a lower SUCRA indicates superior efficacy. SUCRA analysis showed that 1 μg/kg LD then 0.6 μg/kg/h MD (1.4%), 0.4 μg/kg/h IOD (8.5%), and 1 μg/kg LD then 0.3 μg/h MD (20.8%) were the three most effective dosing regimens for lowering postoperative blood IL-6 level, whereas the same volume of NS (89.0%) and 0.3 μg/kg LD then 0.3 μg/kg/h MD (83.5%) were the least effective (Figure 6C).

**Figure 7:**
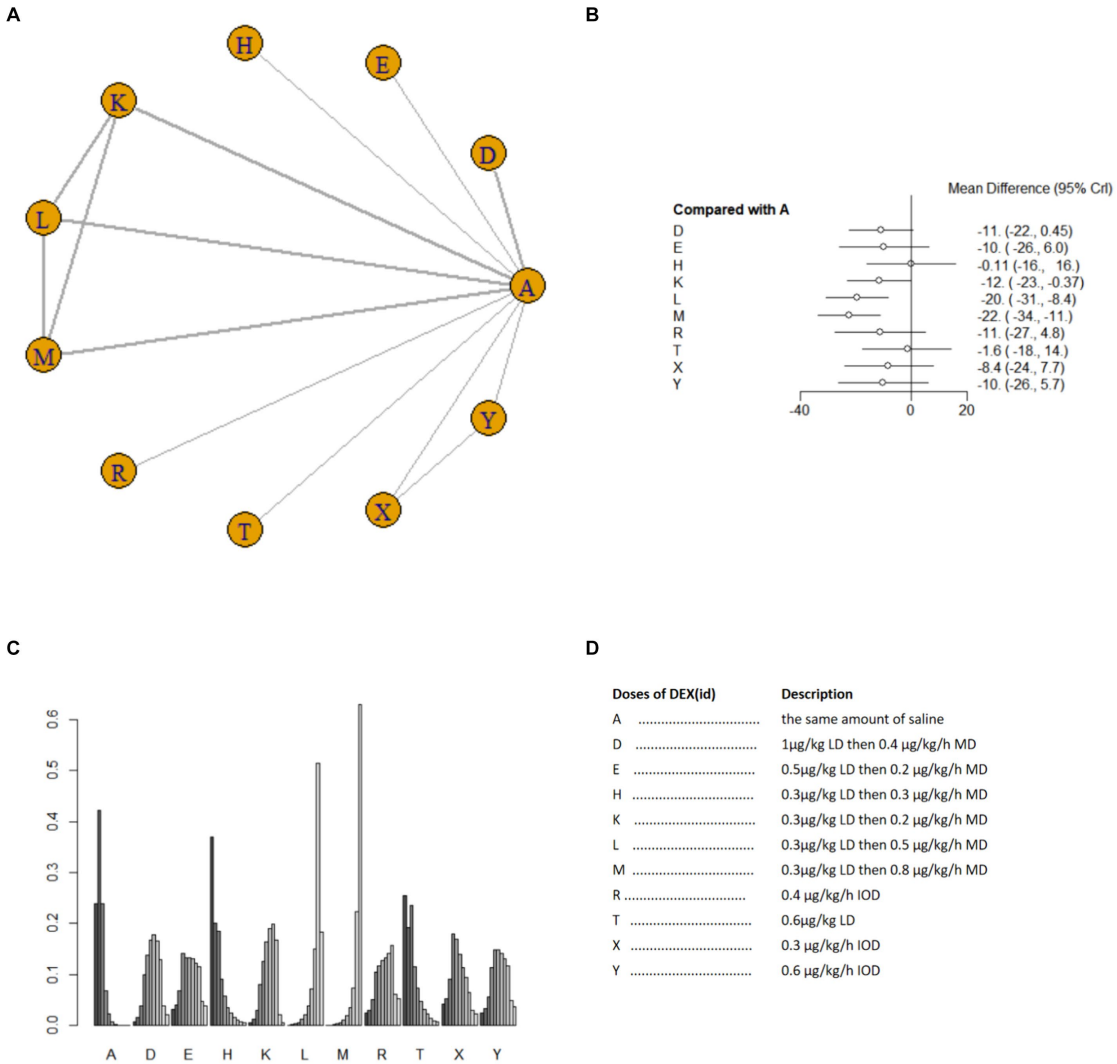
Network estimates of blood TNF-α level after surgery. **(A)** Network plot showing direct comparisons between nodes. **(B)** Forest plot of different DEX doses compared with NS in NMA. **(C)** Estimated probability (%) of each dose level. **(D)** Description of individual DEX doses in this analysis. LD, Load dose; MD, Maintenance dose; IOD, Intraoperative dose.

Nine studies (17, 18, 21, 25, 26, 33–35, 40) involving 1,073 patients reported postoperative blood TNF-α level. A random effects model was used for meta-analysis due to high heterogeneity among studies (*I^2^* = 87%). A total of 10 intervention doses were involved in the NMA, and the evidence relationships are shown in Figure 7A. Compared with the placebo, three DEX dosing regimens 0.3 μg/kg LD then 0.2 μg/kg/h MD,0.3 μg/kg LD then 0.5 μg/kg/h MD, 0.3 μg/kg LD then 0.8 μg/kg/h MD significantly reduced postoperative blood TNF-α level (SMD-12, 95%CI -23 to −0.37; SMD-20, 95%CI -31 to −8.4; SMD -22, 95%CI -34 to −11, respectively) (Figure 7B). Among the different doses, the reduction in postoperative blood TNF-α level was significantly lower with 0.3 μg/kg LD then 0.3 μg/kg/h MD than with 0.3 μg/kg LD then 0.5 μg/kg/h MD and 0.3 μg/kg LD then 0.8 μg/kg/h MD (Supplementary Table 1e). Cumulative ranking of the 12 intervention doses from most effective to least effective based on the SURCA was 0.3 μg/kg LD then 0.8 μg/kg/h MD (6.6%) > 0.3 μg/kg LD then 0.5 μg/kg/h MD (14.2%) > 0.3 μg/kg LD then 0.2 μg/kg/h MD (42.6%) > 0.4 μg/kg/h IOD (43.1%) > 1 μg/kg LD then 0.4 μg/kg/h MD (43.8%) > 0.6 μg/kg/h IOD (46.5%) > 0.5 μg/kg LD then 0.2 μg/kg/h MD (48.4%) > 0.3 μg/kg/h IOD (54.6%) > 0.6 μg/kg LD (78.9%) > 0.3 μg/kg LD then 0.3 μg/kg/h MD (83.1%) > same volume of NS (87.6%) (Figure 7C), see Figure 7D for abbreviations and descriptions of the 10 DEX intervention doses and the control group.

Visual inspection of the comparative adjusted funnel plots did not reveal publication bias in the postoperative day 1, 3, 7 MMSE scores and blood IL-6 lever in our network meta-analysis. The results of Egger’s test (*p* = 0.964, 0.993, 0.339 and 0.126) also negated the presence of a small study effect. Because there were no more than 10 studies on TNF-α, the publication bias analysis did not employ the inverted funnel plot method and the Egger’s test (Supplementary Figures 3a–d).

## 4 Discussion

The results of this study demonstrated that a 1 μg/kg LD followed by a 0.6 μg/kg/h MD was the best dosing regimen for improving postoperative MMSE scores on days 1, 3, and 7. Conversely, an IOD of 0.5 μg/kg/h had the least favorable effect. Additionally, the 1 μg/kg LD followed by 0.6 μg/kg/h MD regimen exhibited the greatest efficacy in reducing postoperative blood IL-6 level, while the 0.3 μg/kg LD followed by a 0.8 μg/kg/h MD regimen was the best approach for lowering postoperative blood TNF-α level. NS had the last effect on postoperative blood levels of IL-6 and TNF-α. Collectively, our findings demonstrate that a 1 μg/kg LD followed by a 0.6 μg/kg/h MD of DEX is superior for reducing postoperative blood IL-6 level and improving postoperative MMSE scores, while caution should be exercised when considering the 0.5 μg/kg/h IOD or 0.3 μg/kg LD then 0.2 μg/kg/h MD regimens. Although the certainty of evidence was low, these results nonetheless indicated that there is an optimal DEX dosing regimen that can improve postoperative cognitive function and postoperative blood IL-6 level. It is interesting to note that the optimal dose converges while the worst dose does not.

Does DEX exert neuroprotective effects through its anti-inflammatory action? DEX inhibits the production and release of key inflammatory mediators, such as cytokines IL-6 and TNF-α (43). These mediators are crucial in the inflammatory response, and their reduction diminishes the intensity and extent of inflammation. DEX influences immune cell function, such as macrophages and neutrophils (44), reducing their accumulation and activation at the inflammation site, thus mitigating the local inflammatory response. By inhibiting inflammatory mediators and reducing oxidative stress, DEX promotes nerve cell survival and decreases apoptosis rates. Inflammatory responses can harm the blood–brain barrier, increasing its permeability and worsening neurological damage. Dexmedetomidine’s anti-inflammatory effect helps maintain and restore the blood–brain barrier’s integrity (45), preventing harmful substances from entering brain tissue. These pathways demonstrate DEX’s significant neuroprotective and nerve repair effects, with its anti-inflammatory action being a crucial mechanism. Further research could delve into these mechanisms and assess DEX’s therapeutic potential in various neurological disorders.

We included studies in which DEX was used in different surgical and anaesthesia modalities. Our meta-analysis showed that DEX improves neurological outcomes in different surgical and anesthesia approaches, consistent with previous studies. A study comparing pre-anesthetic doses of ketamine and DEX in older patients undergoing emergency surgery showed promising results in reducing cognitive dysfunction (46). In addition, a study assessing the impact of DEX combined with etomidate on postoperative cognitive function in older patients undergoing ureteroscopic holmium laser lithotripsy revealed decreased incidences of cognitive dysfunction, emergent agitation, depression, and anxiety (47). Furthermore, a recent network meta-analysis involving 24 RCTs reported that intravenous DEX infusion during non-cardiac and non-neurosurgical procedures led to significantly reduced incidences of postoperative delirium (POD) and cognitive deficits when compared to alternative interventions (1). DEX has also demonstrated neuroprotective effects across different anesthesia modalities. It was found that the incidence of POD was lower in patients using DEX in spinal anaesthesia than in those using propofol (48). Additionally, a meta-analysis of 18 RCTs suggested that intravenous DEX sedation during surgery could play a crucial role in preventing POD and cognitive dysfunction in older patients (≥ 60 years old) receiving regional anesthesia. Moreover, transnasal administration of DEX has been shown to be effective for improving sleep disturbances, preventing neurocognitive impairment and POD, reducing anxiety, and minimizing adverse effects and complications in older patients undergoing general anaesthesia (49). Taken together, DEX is a promising sedative for enhancing cognitive function in various surgical procedures, anaesthesia modalities, and administration routes.

DEX has demonstrated favourable outcomes in postoperative sedation (50), pain management, anxiety reduction (51), cognitive function, and postoperative inflammation. Additionally, it has a unique and manageable mechanism of action that results in a low risk of respiratory depression (52) compared to other sedatives. However, DEX can also pose several adverse effects, including hypotension (53), bradycardia (54), and over-sedation. These risks may vary between different patient populations and should be closely monitored in the clinical setting.

Due to the presence of potential heterogeneity, the ranking of DEX doses in the NMA may not be directly applicable to future clinical treatment rankings. Therefore, our findings should be interpreted with caution, and randomized-controlled trials will be required to directly compare the effect of different DEX doses on postoperative cognitive function and inflammatory response.

This study has two strengths. First, this is the first and largest systematic review using a NMA approach to determine the effect of different DEX doses on postoperative cognitive function and inflammatory markers. The findings of this work provide valuable insight into the optimal DEX dosing regimen for reducing the incidences of postoperative cognitive dysfunction and postoperative inflammation. Second, the results of this systematic review and NMA can serve as support for future studies on the same topic.

Several limitations should be noted for this study. Differences in study design between RCTs and retrospective comparative studies (RCS) can impact result interpretation, since the latter is prone to selection bias and information bias due to its non-randomized nature. Additionally, high heterogeneity could indicate variations in populations, interventions, and outcome measures, which may impact result stability and interpretation. Lastly, potential limitations in data availability and study quality should be considered. Nonetheless, further large-cohort RCTs are warranted to minimize heterogeneity, improve internal validity, and assess the long-term safety and tolerability of DEX in different patient populations.

## 5 Conclusion

In conclusion, our NMA demonstrates the benefits of perioperative DEX use in improving postoperative cognitive function and postoperative inflammatory response. In addition, a 1 μg/kg LD then 0.6 μg/kg/h MD of DEX is the optimal dosing regimen for improving postoperative cognitive function and lowering blood IL-6 levels, whereas a 0.3 μg/kg LD then 0.2 μg/kg/h MD is the best regimen for lowering postoperative blood TNF-α level. Due to the limitations of this study and the heterogeneity among the included studies, these findings should be interpreted with caution. Nevertheless, we hope that our findings will provide valuable insights into the selection of appropriate DEX regimens for elective surgery patients.

## Data availability statement

The original contributions presented in the study are included in the article/Supplementary material, further inquiries can be directed to the corresponding author.

## Author contributions

CH: Conceptualization, Data curation, Formal analysis, Methodology, Software, Visualization, Writing – original draft, Writing – review & editing. RY: Data curation, Methodology, Writing – review & editing, Investigation, Supervision. XX: Investigation, Methodology, Writing – original draft. HD: Conceptualization, Resources, Supervision, Validation, Writing – review & editing. LP: Conceptualization, Data curation, Project administration, Resources, Supervision, Validation, Writing – review & editing.
