# Supplementary material for: Effects of dexmedetomidine on early postoperative cognitive function and postoperative inflammatory response: a systematic review and network meta-analysis
Source: Front Neurol. 2024 Aug 12;15:1422049. doi: 10.3389/fneur.2024.1422049 (PMC11346340; doi:10.3389/fneur.2024.1422049)
Supplement: Supplementary file 1 [file Table_1.DOCX]

**Appendix A**

**Embase session results**

| No. | Query |
| --- | --- |
| #8 | #7 AND 'Article'/it |
| #7 | #3 AND #6 |
| #6 | #4 OR #5 |
| #5 | 'cognition disorder' OR 'cognition disorders' OR 'cognitive defects' OR 'cognitive deficit' OR 'cognitive disability' OR 'cognitive disorder' OR 'cognitive disorders' OR 'cognitive dysfunction' OR 'cognitive impairment' OR 'delirium, dementia, amnestic, cognitive disorders' OR 'overinclusion' OR 'response interference' OR 'cognitive defect' |
| #4 | 'cognitive defect'/exp |
| #3 | #1 OR #2 |
| #2 | 'bxcl 501' OR 'bxcl501' OR 'cepedex' OR 'da 9501' OR 'da9501' OR 'delos' OR 'dexamedetomidine' OR 'dexdomitor' OR 'dexdor' OR 'dexmedetomidine hydrochloride' OR 'igalmi' OR 'mpv 1440' OR 'mpv1440' OR 'precedex' OR 'primadex' OR 'sedadex' OR 'sileo' OR 'tpu 006' OR 'tpu006' OR 'dexmedetomidine' |
| #1 | 'dexmedetomidine'/exp |

**Pubmed session results**

| Search number | Query |
| --- | --- |
| 5 | (((("Postoperative Cognitive Complications"[Mesh] OR(Postoperative Cognitive Complications)OR (postoperative cognitive dysfunction) OR (Postoperative Cognitive Complication) OR (Postoperative Cognitive Dysfunction) OR (Postoperative Cognitive Decline) OR (Postoperative Dementia) OR (Postoperative Cognitive Disorders)) AND ("Dexmedetomidine"[Mesh] OR (MPV1440) OR (Precedex) OR(Dexdomitor) OR (Sedadex) OR(Sileo) OR(Cepedex) OR (Dexdor) OR (Dexmedetomidine Hydrochloride) OR (Igalmi) OR'bxcl 501' OR 'bxcl501' OR 'cepedex' OR 'da 9501' OR 'da9501' OR 'delos' OR 'dexamedetomidine' OR 'dexdomitor' OR 'dexdor' OR 'dexmedetomidine hydrochloride' OR 'igalmi' OR 'mpv 1440' OR 'mpv1440' OR 'precedex' OR 'primadex' OR 'sedadex' OR 'sileo' OR 'tpu 006' OR 'tpu006' OR 'dexmedetomidine')) ) NOT (letter[Publication Type])) NOT (review[Publication Type]) |
| 4 | ("Postoperative Cognitive Complications"[Mesh] OR(Postoperative Cognitive Complications)OR (postoperative cognitive dysfunction) OR (Postoperative Cognitive Complication) OR (Postoperative Cognitive Dysfunction) OR (Postoperative Cognitive Decline) OR (Postoperative Dementia) OR (Postoperative Cognitive Disorders)) AND ("Dexmedetomidine"[Mesh] OR (MPV1440) OR (Precedex) OR(Dexdomitor) OR (Sedadex) OR(Sileo) OR(Cepedex) OR (Dexdor) OR (Dexmedetomidine Hydrochloride) OR (Igalmi) OR'bxcl 501' OR 'bxcl501' OR 'cepedex' OR 'da 9501' OR 'da9501' OR 'delos' OR 'dexamedetomidine' OR 'dexdomitor' OR 'dexdor' OR 'dexmedetomidine hydrochloride' OR 'igalmi' OR 'mpv 1440' OR 'mpv1440' OR 'precedex' OR 'primadex' OR 'sedadex' OR 'sileo' OR 'tpu 006' OR 'tpu006' OR 'dexmedetomidine') |
| 3 | "Postoperative Cognitive Complications"[Mesh] OR(Postoperative Cognitive Complications)OR (postoperative cognitive dysfunction) OR (Postoperative Cognitive Complication) OR (Postoperative Cognitive Dysfunction) OR (Postoperative Cognitive Decline) OR (Postoperative Dementia) OR (Postoperative Cognitive Disorders) |
| 2 | "Cognition Disorders"[Mesh] OR(Disorder, Cognition )OR (Overinclusion) OR'cognition disorder' OR 'cognition disorders' OR 'cognitive defects' OR 'cognitive deficit' OR 'cognitive disability' OR 'cognitive disorder' OR 'cognitive disorders' OR 'cognitive dysfunction' OR 'cognitive impairment' OR 'delirium, dementia, amnestic, cognitive disorders' OR 'overinclusion' OR 'response interference' OR 'cognitive defect' |
| 1 | "Dexmedetomidine"[Mesh] OR (MPV1440) OR (Precedex) OR(Dexdomitor) OR (Sedadex) OR(Sileo) OR(Cepedex) OR (Dexdor) OR (Dexmedetomidine Hydrochloride) OR (Igalmi) OR'bxcl 501' OR 'bxcl501' OR 'cepedex' OR 'da 9501' OR 'da9501' OR 'delos' OR 'dexamedetomidine' OR 'dexdomitor' OR 'dexdor' OR 'dexmedetomidine hydrochloride' OR 'igalmi' OR 'mpv 1440' OR 'mpv1440' OR 'precedex' OR 'primadex' OR 'sedadex' OR 'sileo' OR 'tpu 006' OR 'tpu006' OR 'dexmedetomidine' |

**Search strategy of Cochrane Library**

ID Search Hits

#1 MeSH descriptor: [Dexmedetomidine] explode all trees

#2 (bxcl 501):ti,ab OR (bxcl501):ti,ab OR (cepedex):ti,ab OR (da 9501):ti,ab OR (da9501):ti,ab OR (delos):ti,ab OR (dexamedetomidine):ti,ab OR (dexdomitor):ti,ab OR (dexdor):ti,ab OR (dexmedetomidine hydrochloride):ti,ab OR (igalmi):ti,ab OR (mpv 1440):ti,ab OR (mpv1440):ti,ab OR (precedex):ti,ab OR (primadex):ti,ab OR (sedadex):ti,ab OR (sileo):ti,ab OR (tpu 006):ti,ab OR (tpu006):ti,ab OR (dexmedetomidine):ti,ab,kw

#3 #1 or #2

#4 MeSH descriptor: [Postoperative Cognitive Complications] explode all trees 2774

#5 (Postoperative Cognitive Complications):ti,ab,kw OR (postoperative cognitive dysfunction):ti,ab,kw OR (Postoperative Cognitive Complication):ti,ab,kw OR (Postoperative Cognitive Dysfunction):ti,ab,kw OR (Postoperative Cognitive Decline):ti,ab,kw OR (Postoperative Dementia):ti,ab,kw OR (Postoperative Cognitive Disorders):ti,ab,kw

#6 #4 or #5

#7 #3 AND #6

**Supplementary Table 1a** MMSE1 score - indirect comparison of different dose


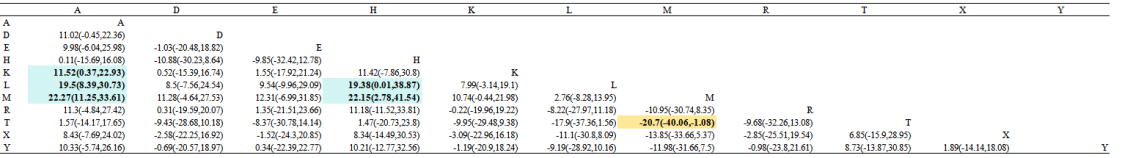


Mean change based on baseline was analysed. Positive standardised mean differences indicate improved MMSE scores on the first postoperative day. Statistically significant changes are indicated using bold and colour (blue indicates row treatments significantly better than column treatments) and white indicates no statistically significant difference between treatments. A,the same amount of saline; D,1μg/kg load then 0.4μg/kg/h; E,0.5μg/kg load then 0.2μg/kg/h; H,0.3μg/kg load then 0.3μg/kg/h; K,0.3μg/kg load then 0.2 μg/kg/h; L,0.3μg/kg load then 0.5μg/kg/h; M,0.3μg/kg load then 0.8μg/kg/h; R,0.3μg/kg load then 0.8 μg/kg/h; T,0.6μg/kg load; X,intraoperative 0.3 μg/kg/h; Y,intraoperative 0.6μg/kg/h.

**Supplementary Table 1b** MMSE3 score - indirect comparison of different dose


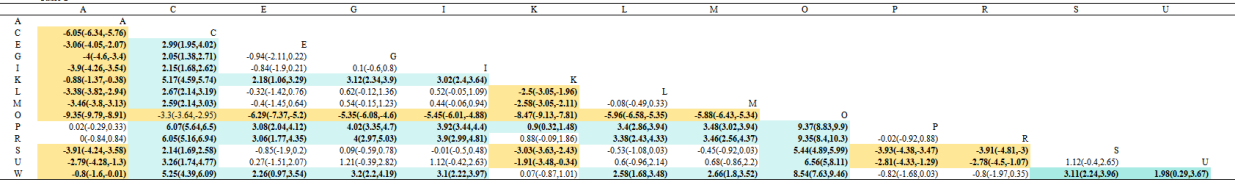


Mean changes based on baseline were analysed. A positive standardised mean difference indicates an improvement in MMSE score on the third postoperative day. Statistically significant changes are indicated in bold and colour (blue indicates row treatment significantly better than column treatment), and white indicates no statistically significant difference between treatments.A,the same amount of saline; C,1μg/kg load then 0.3μg/h; E,0.5μg/kg load then 0.2μg/kg/h; G,1μg/kg load then 0.3-0.5μg/kg/h; I,1μg/kg load then 0.2 μg/kg/h; K,0.3μg/kg load then 0.2μg/kg/h; L,0.3μg/kg load then 0.5 μg/kg/h; M,0.3μg/kg load then 0.8μg/kg/h; O,1μg/kg load then 0.6μg/kg/h; P,intraoperative 0.5 µg/kg/h; R,intraoperative 0.4 µg/kg/h; S,1μg/kg then 0.5μg/kg/h; U,0.5 µg/kg on thirty minutes before the end of surgery; W,0.8μg/kg load then 0.2 μg/kg/h.

**Supplementary Table 1c** MMSE7 score - indirect comparison of different dose


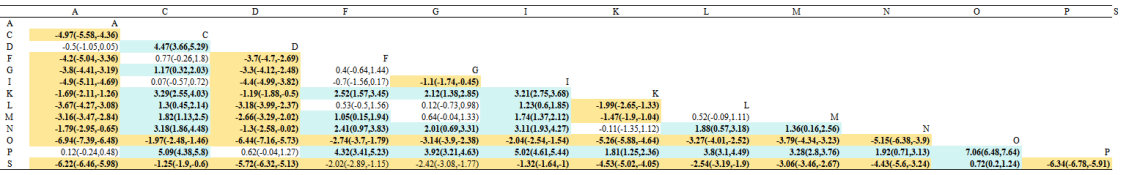


Mean changes based on baseline were analysed. A positive standardised mean difference indicates an improvement in MMSE score on the seventh postoperative day. Statistically significant changes are indicated in bold and colour (blue indicates row treatment significantly better than column treatment), and white indicates no statistically significant difference between treatments.A,the same amount of saline; C,1μg/kg load then 0.3μg/h; D,1μg/kg load then 0.4μg/kg/h; F,0.6μg/kg load then 0.2μg/kg/h; G,1μg/kg load then 0.3-0.5μg/kg/h; I,1μg/kg load then 0.2μg/kg/h; K,0.3μg/kg load then 0.2μg/kg/h; L,0.3μg/kg load then 0.5μg/kg/h; M,0.3μg/kg load then 0.8μg/kg/h; N,0.5μg/kg load then 0.6μg/kg/h; O,1μg/kg load then 0.6μg/kg/h; P,intraoperative 0.5 µg/kg/h; S,1μg/kg then 0.5μg/kg/h.

**Supplementary Table 1d** IL-6 lever - indirect comparison of different dose


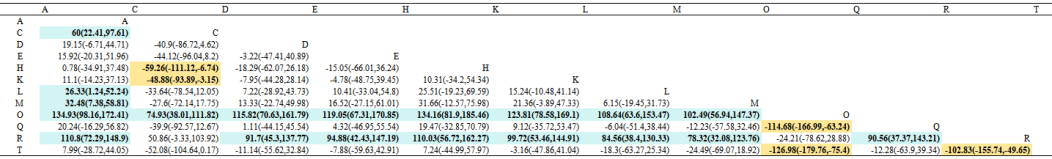


Mean changes based on baseline were analysed. A positive standardised mean difference indicates an increase in plasma IL-6 levels after surgery. Statistically significant changes are indicated in bold and colour (blue indicates row treatment significantly better than column treatment) and white indicates no statistically significant difference between treatments.A,the same amount of saline; C,1μg/kg load then 0.3μg/h; D,1μg/kg load then 0.4μg/kg/h; E,0.5μg/kg load then 0.2μg/kg/h; H,0.3μg/kg load then 0.3μg/kg/h; K,0.3μg/kg load then 0.2μg/kg/h; L,0.3μg/kg load then 0.5μg/kg/h; M,0.3μg/kg load then 0.8μg/kg/h; O,1μg/kg load then 0.6μg/kg/h; Q,0.8μg/kg load then 0.5μg/kg/h; R,intraoperative 0.4 µg/kg/h; T,0.6μg/kg load.

**Supplementary Table 1e** TNF-α lever - indirect comparison of different dose


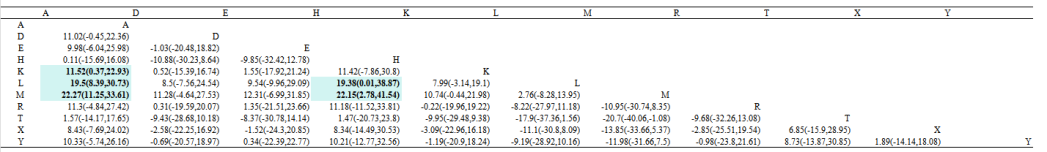


Mean changes based on baseline were analysed. A positive standardised mean difference indicates an increase in plasma TNF-α levels after surgery. Statistically significant changes are indicated in bold and colour (blue indicates row treatment significantly better than column treatment) and white indicates no statistically significant difference between treatments.A,the same amount of saline; D,1μg/kg load then 0.4μg/kg/h; E,0.5μg/kg load then 0.2μg/kg/h; H,0.3μg/kg load then 0.3μg/kg/h; K,0.3μg/kg load then 0.2μg/kg/h; L,0.3μg/kg load then 0.5μg/kg/h; M,0.3μg/kg load then 0.8μg/kg/h; R,intraoperative 0.4µg/kg/h; T,0.6μg/kg load; X,intraoperative 0.3μg/kg/h; Y,intraoperative 0.6μg/kg/h.

| a | b |
| --- | --- |
| 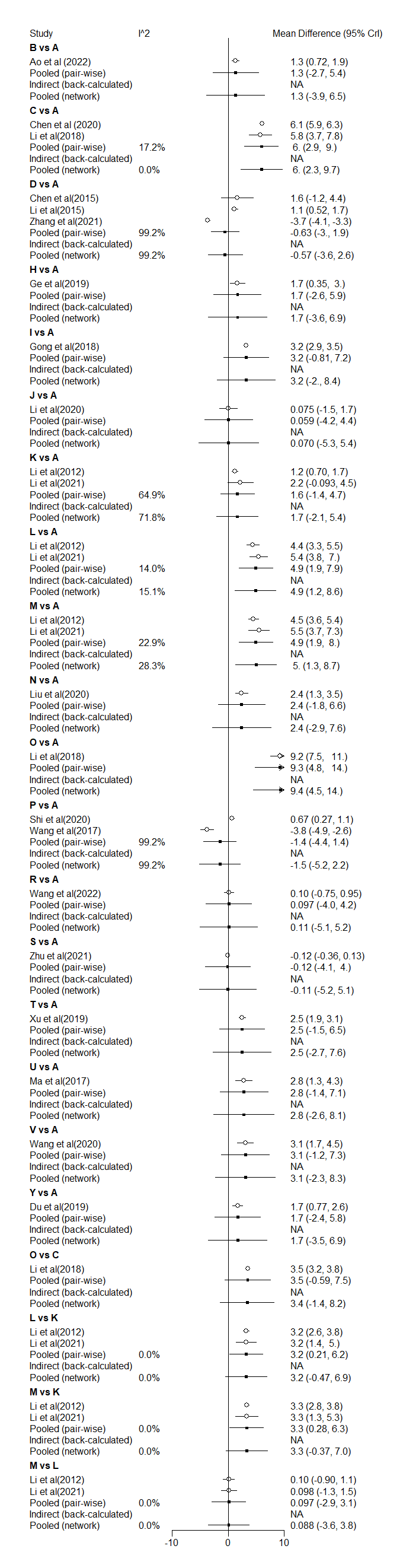 | 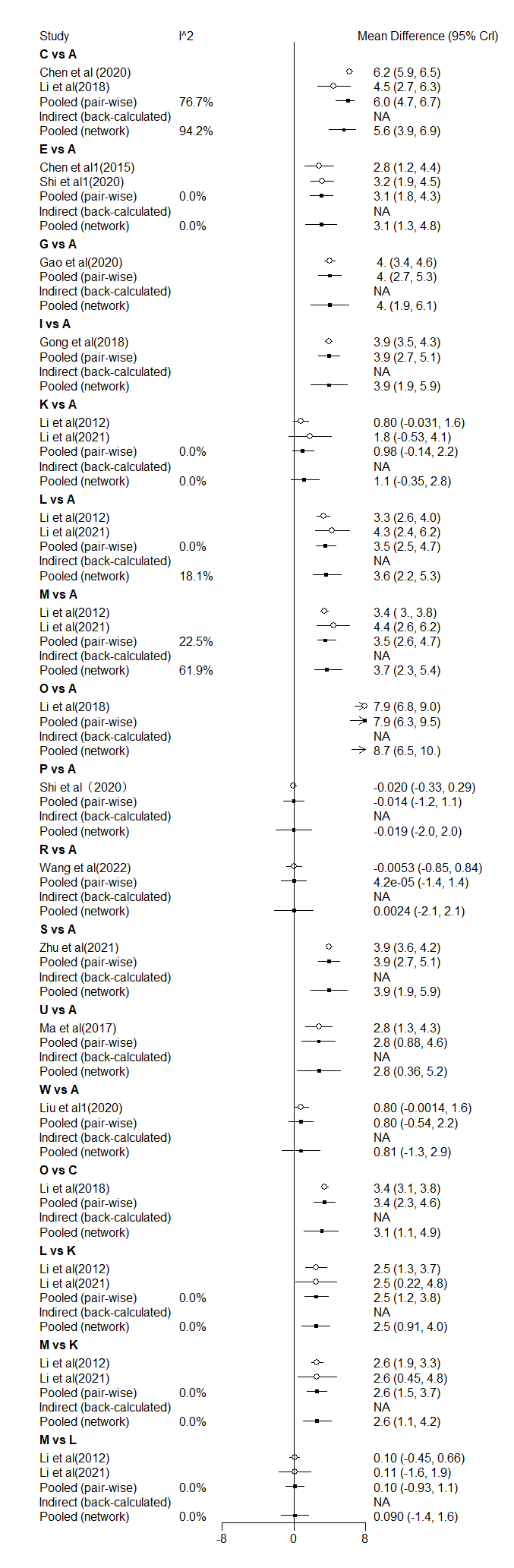 |
| c | d |
| 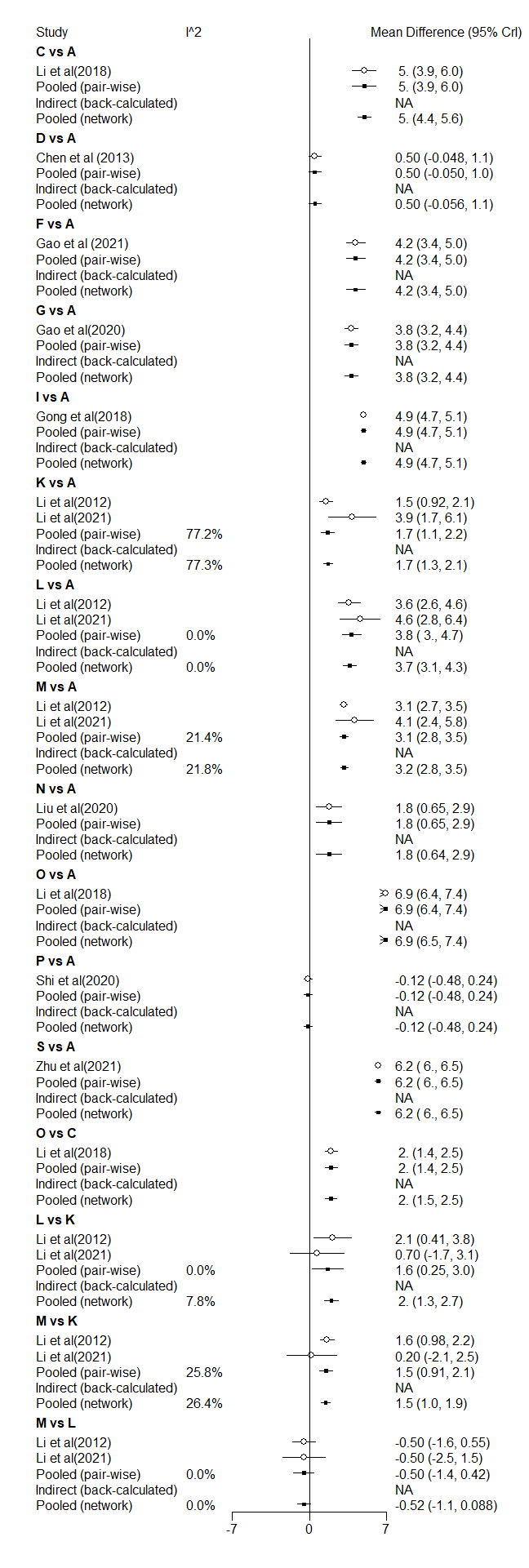 | 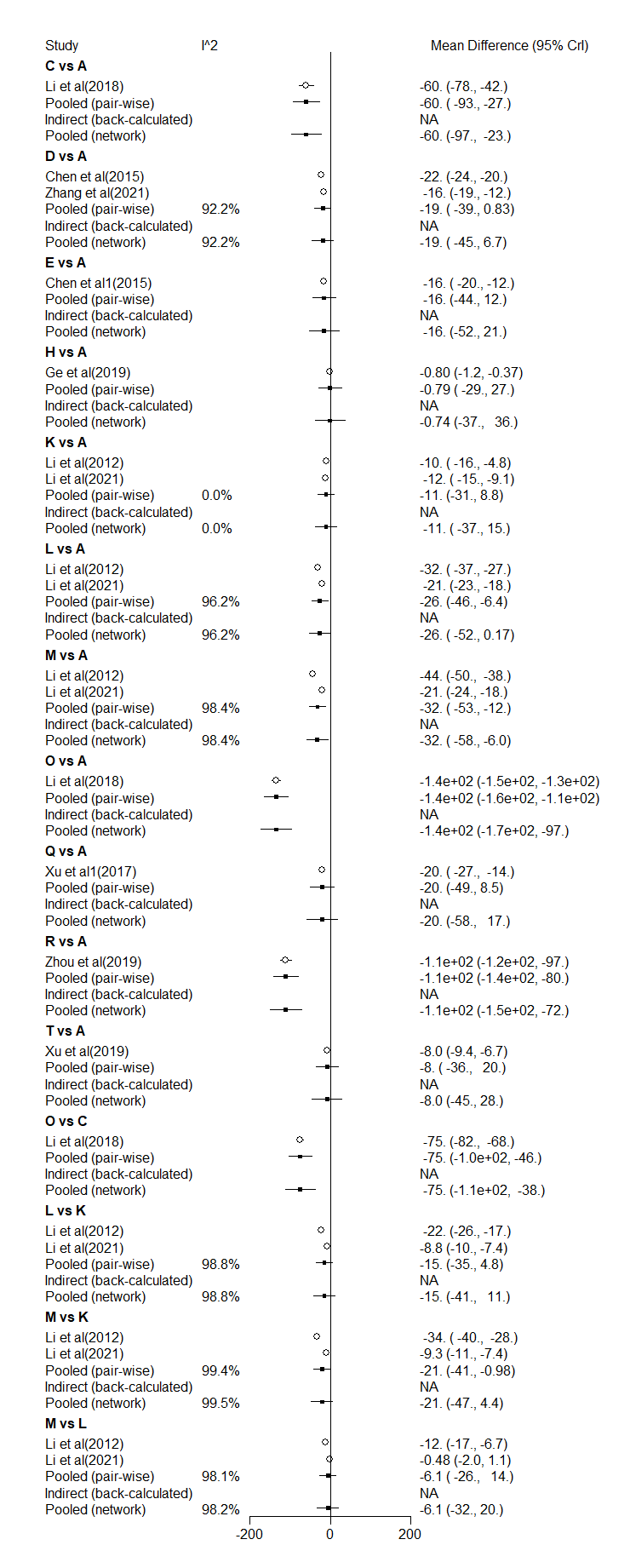 |
| e | f |
| 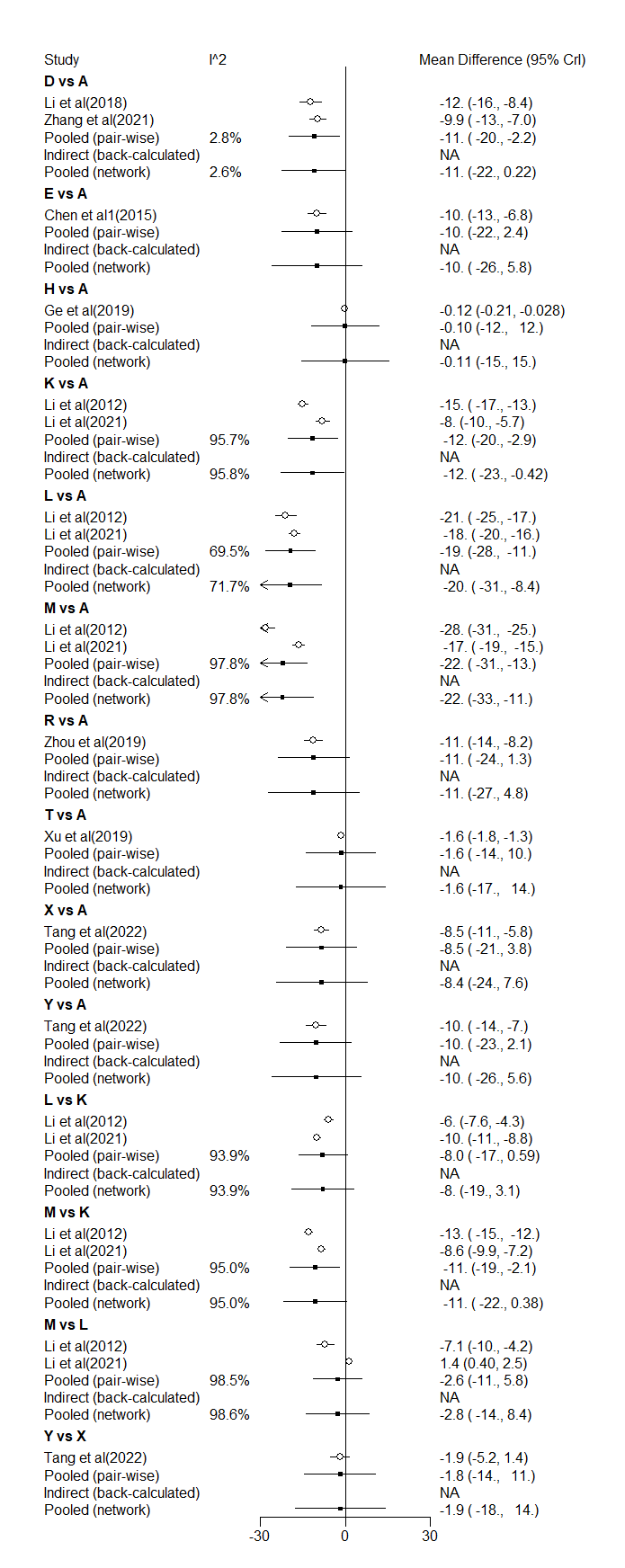 | 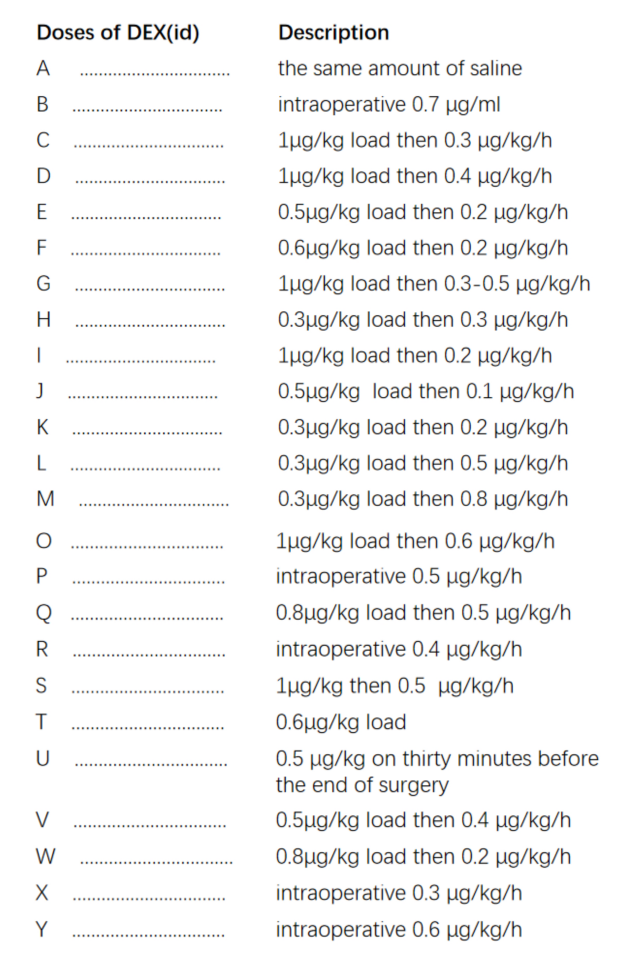 |

**Supplementary Figure 1**. Results of network meta-analysis of MMSE scores and inflammatory factors after treatment with different doses of dexmedetomidine. Network meta-analysis of different doses of dexmedetomidine on MMSE scores on postoperative day 1 (a),day 3 (b),day 7 (c), IL-6 (d) and TNF-α (e). (f) Description of individual doses of dexmedetomidine in this analysis.

| 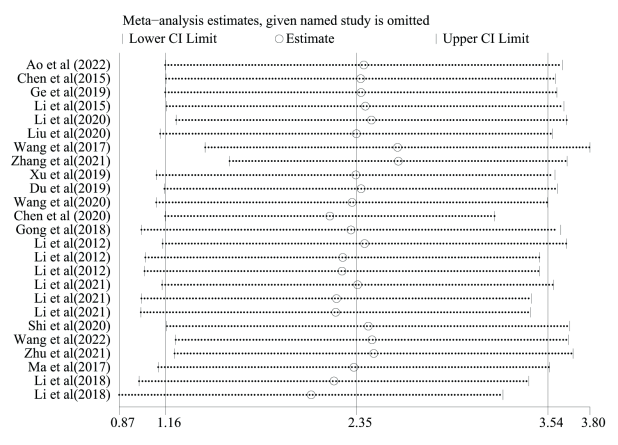  a | 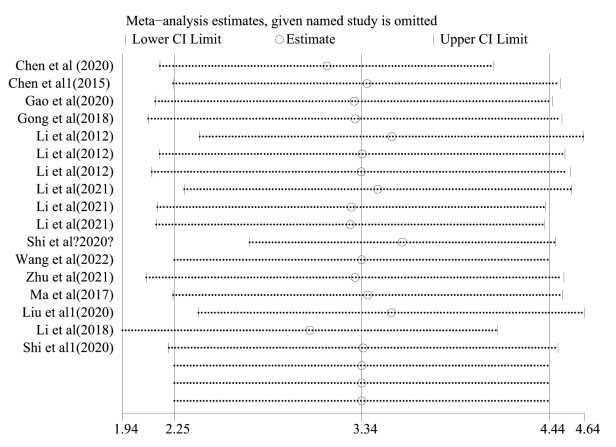  b |
| --- | --- |
| 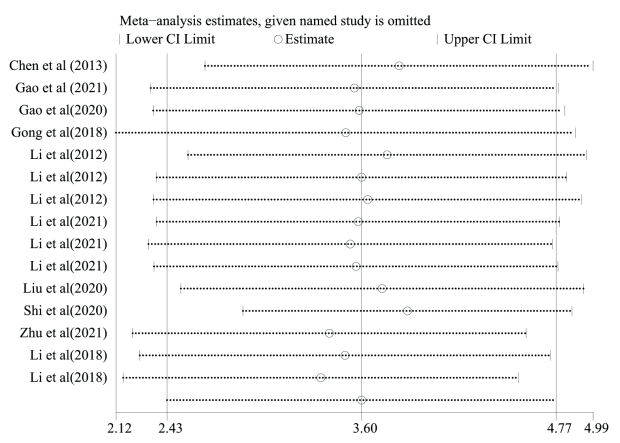  c | 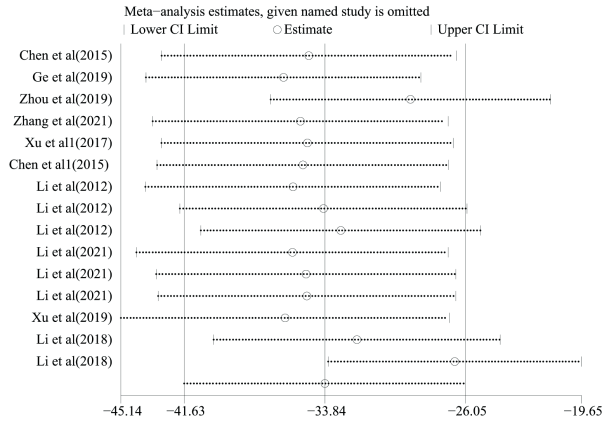  d |
| 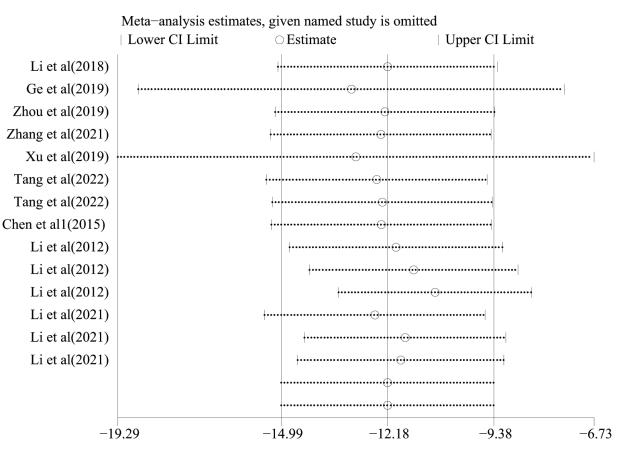  e |  |

**Supplementary Figure 2.** Sensitivity analysis of NMA of MMSE scores and inflammatory factors.Sensitivity analysis of MMSE score on postoperative day 1(a), day 3(b),day 7(c), Funnel plot of postoperative blood IL-6 (d) and TNF-α(e) levels.

| 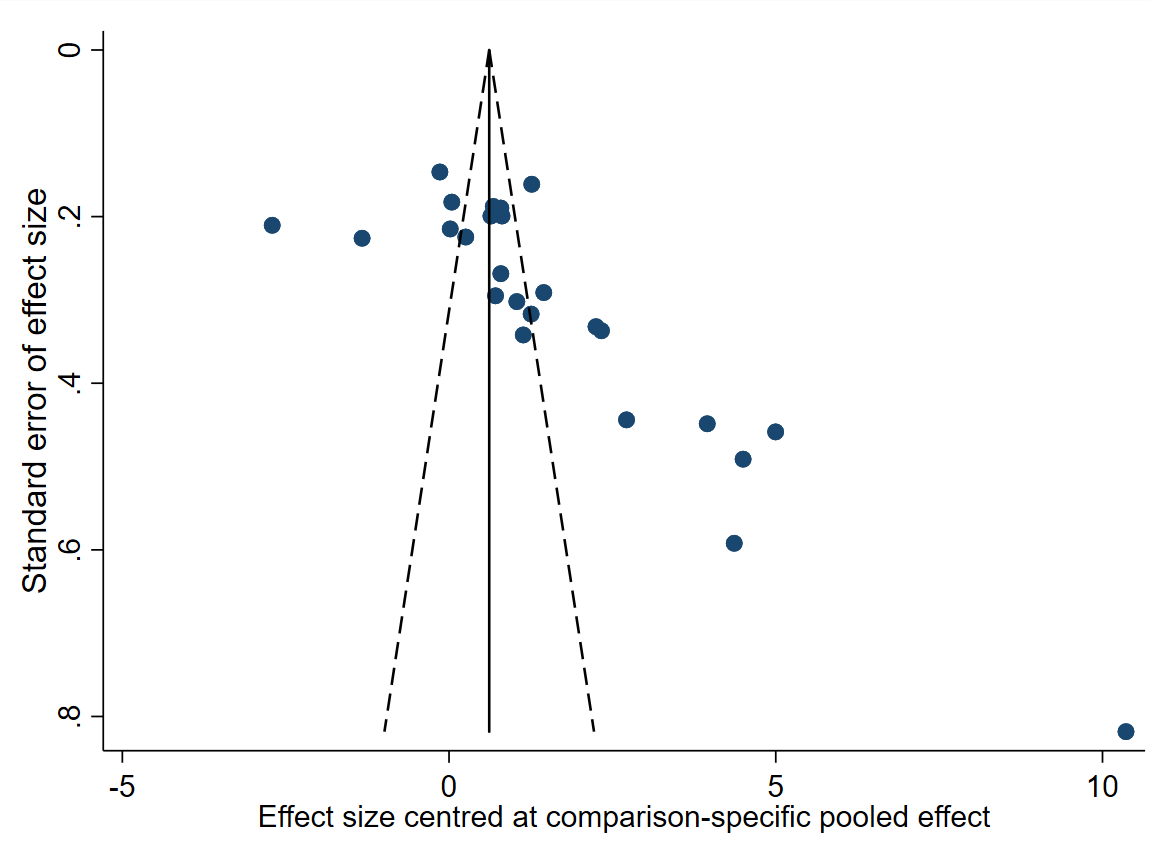  a | 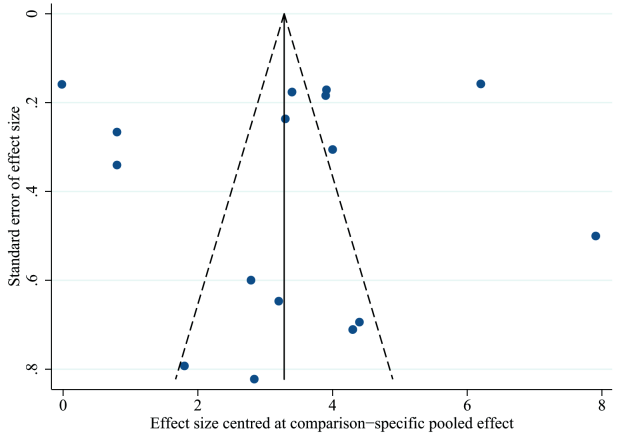  b |
| --- | --- |
| 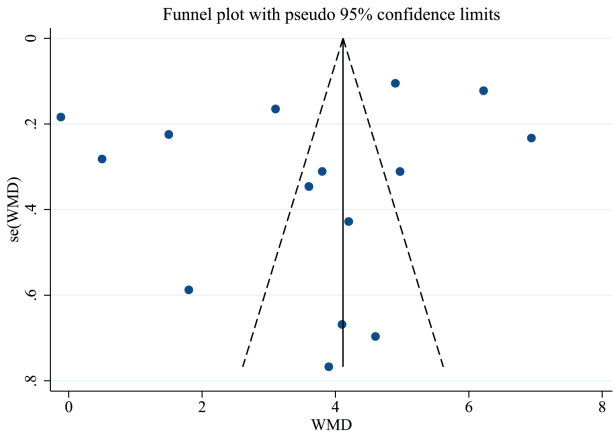  c | 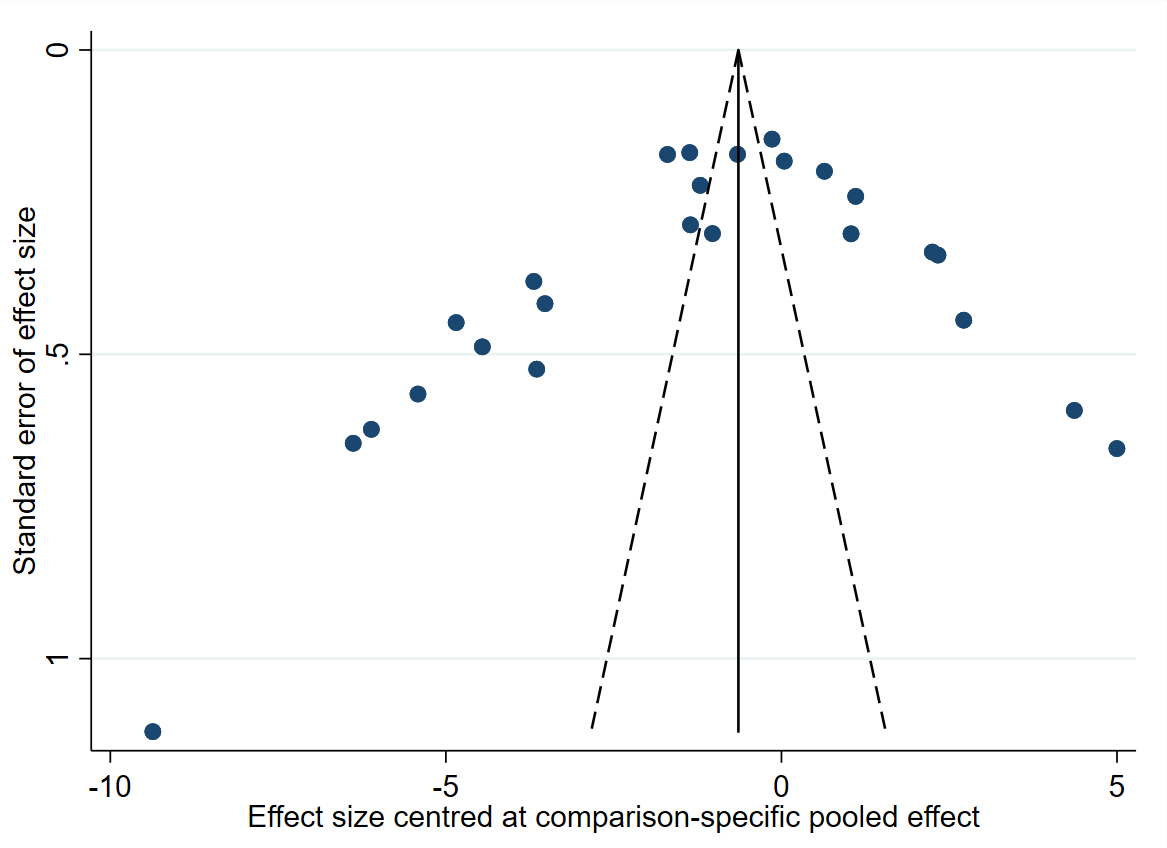  d |

**Supplementary Figure 3.** Funnel plot of NMA of MMSE scores and inflammatory factors. Funnel plot of MMSE score on postoperative day 1(a), day 3(b),day 7(c), Funnel plot of postoperative blood IL-6 levels(d).
